# Distinct functional heterogeneity of TP53 R175 mutations in platinum-resistant ovarian cancer: unveiling molecular mechanisms and therapeutic targets

**DOI:** 10.1038/s41419-025-08172-0

**Published:** 2025-11-17

**Authors:** Yufeng Liu, Zhiguo Zheng, Maowei Ni, Shuyu Mao, Yue Xiao, Ye Zhao, Bing Tian, Liangyan Wang, Hong Xu, Yuejin Hua

**Affiliations:** 1 MOE Key Laboratory of Biosystems Homeostasis & Protection, Institute of Biophysics, College of Life Science, Zhejiang University, Hangzhou, China; 2 Zhejiang Cancer Hospital, Hangzhou Institute of Medicine (HIM), Chinese Academy of Sciences, Hangzhou, China; 3 Cancer Center, Zhejiang University, Hangzhou, China

**Keywords:** Cancer, Cancer

## Abstract

Ovarian cancer (OC) is a highly aggressive malignancy in women, and platinum resistance remains a major clinical obstacle. p53 mutations are prevalent in OC and exhibit functional heterogeneity that is associated with therapeutic response and disease progression. However, the roles and mechanisms underlying the functional heterogeneity of p53 mutations in platinum-resistant OC remain elusive. This investigation delineated that p53 mutations within the Loop 2, Loop 3, and β-strand S10 regions were closely linked to platinum resistance. In particular, functional assays unveiled that p53^R175H^ and p53^R175G^ mutations at Arg175 revealed distinct roles in tumor cell migration and drug resistance, with p53^R175G^ conferring resistance to agents targeting p53^R175H^. Through multi-omics sequencing analysis, it was discerned that p53^R175H^ and p53^R175G^ promoted tumor progression through distinct cofactors and regulatory networks. p53^R175H^ mediated upregulation of extracellular matrix-related genes, whereas p53^R175G^ activated pathways associated with cytokine receptor interaction and membrane trafficking. Notably, the chromatin remodeling protein CHD1 selectively interacted with p53^R175G^, but not p53^R175H^, and regulated the transcriptional activity of p53^R175G^, including target genes such as *IL7R*. Moreover, CHD1 knockdown or pharmacological inhibition of IL7R synergistically enhanced platinum sensitivity, suggesting promising combination therapies specifically targeting the R175G mutation. The findings revealed that p53 mutations at the same residue exhibited distinct functional properties and relied on unique cofactors, offering valuable insights for precision therapy in OC.

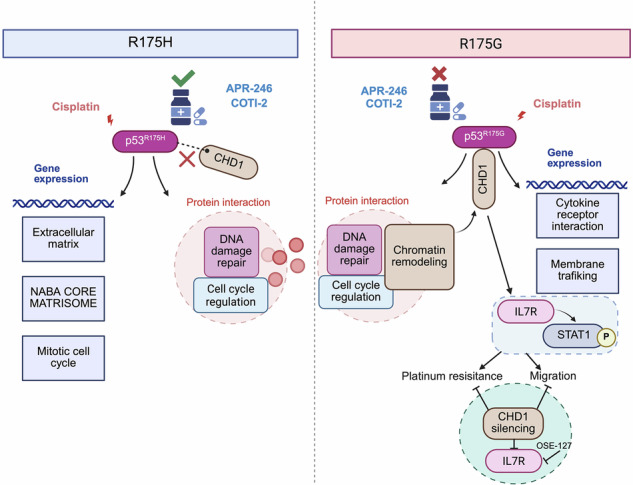

## Introduction

Ovarian cancer (OC) is among the most lethal malignancies affecting the female reproductive system globally [1, 2]. Cytoreductive surgery combined with platinum-based chemotherapy remains the first-line standard of care [3]. Despite initial therapeutic responses, over 80% of patients experience disease recurrence [4], with nearly all eventually developing platinum resistance. For patients with platinum-resistant recurrence, effective treatment options are extremely limited, and the median progression-free survival is less than 1 year [5], underscoring the urgent need for innovative precision therapies.

High-throughput sequencing technologies have characterized the mutational landscape of OC. TP53 is the most frequently mutated gene in OC, with a mutation rate exceeding 80% [6, 7]. The majority of TP53 mutations are missense, with over 2000 identified, primarily concentrated in the DNA-binding domain (DBD) [8–11]. Emerging evidence has demonstrated substantial functional and prognostic heterogeneity among different p53 hotspot mutations, suggesting mutation-specific influences on therapeutic response and drug selection [12, 13]. However, the roles and mechanisms underlying the functional heterogeneity of p53 mutations in platinum-resistant recurrent OC require further elucidation, which is essential for advancing the development of personalized precision medicine strategies.

The transcription factor p53 plays a crucial role in suppressing tumor proliferation by inducing cell cycle arrest, senescence, and apoptosis [14, 15]. TP53 mutations result in a loss of p53’s tumor-suppressor function (LOF), thereby contributing to tumorigenesis in both humans and mice [16, 17]. Furthermore, mutant p53 exhibits oncogenic gain-of-function (GOF) properties, reprogramming the tumor microenvironment (TME) to promote immunosuppression, immune evasion, and therapeutic resistance, thereby accelerating tumor progression [18, 19]. Platinum resistance is a multifactorial process involving enhanced DNA repair, drug efflux pump activation, apoptotic suppression, cancer stem cell maintenance, and TME reprogramming [20–22]. An increasing body of evidence has demonstrated that mutant p53 influences tumor metastasis, epithelial-mesenchymal transition, and other biological processes [23–25]. Mutant p53 can alter genomic functions through interactions with transcription factors, mediation of chromatin remodeling, and acquiring novel DNA-binding activities [26–31]. Moreover, different p53 mutations may exhibit distinct GOF activities, and even an identical mutation may function through diverse mechanisms depending on the cellular context [13, 32–34], further increasing the complexity of the TP53 mutation spectrum.

Funk et al. systematically mapped the functional spectrum of TP53 missense mutations, revealing unique LOF and GOF effects among different mutations, even within the same residue. In addition, targeted therapies developed for the R175H mutation, such as APR-246 and ZMC1, have demonstrated limited efficacy in restoring the tumor-suppressive function in other R175 variants [11]. These limitations underscore the urgent need for alternative therapeutic strategies targeting non-R175H variants at Arg175. Recent studies have increasingly demonstrated the pronounced functional heterogeneity among mutation variants at the same TP53 residue [11, 35, 36]. Given the significant prognostic value of p53 mutations and their demonstrated impact on therapeutic response and drug selection [25], targeting p53 mutations represents a promising strategy to overcome platinum resistance in OC. However, the functional heterogeneity of p53 mutations necessitates further elucidation of their roles and underlying mechanisms in platinum-resistant recurrence of OC.

In this study, we investigated the association between TP53 missense mutations and platinum resistance in OC, with particular emphasis on the functional heterogeneity and molecular mechanisms distinguishing the Arg175 variants (R175H and R175G) in driving platinum resistance. This work aimed to elucidate the therapeutic implications of p53 mutation heterogeneity for individualized treatment, thereby providing new perspectives for advancing personalized precision medicine in OC.

## Results

### TP53 missense mutations contribute to platinum resistance in ovarian cancer

**Fig. 1:**
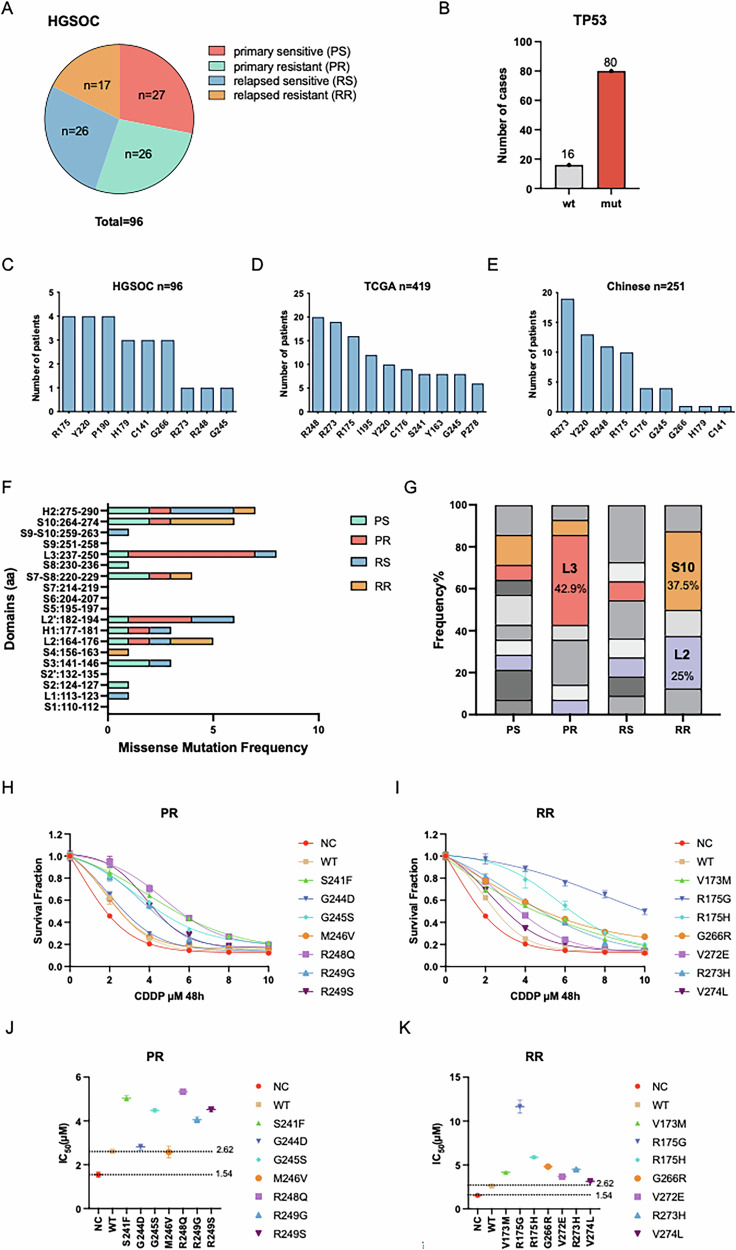
Domain-specific TP53 missense mutations are associated with platinum resistance in OC. **A** 96 HGSOC patients were categorized into four groups: primary-sensitive, primary-resistant, relapse-sensitive, and relapse-resistant. **B** Somatic mutations of TP53. **C–E** TP53 hotspot mutations of ovarian cancer identified in this study, TCGA, and the Chinese cohort. **F** The frequency of TP53 missense mutations within the DBD. **G** The proportion of missense mutations in each secondary domain across the four groups. The Loop 3 (L3), β-strand S10 (S10), and Loop 2 (L2) domains were highlighted in color, while other regions were represented in gray. **H**, **I** CCK-8 assay validating the cisplatin sensitivity of the mutations in the primary-resistant and relapse-resistant groups. The data are presented as a nonlinear fit. **J**, **K** 50% inhibitory concentration (IC_50_, with 95% CI) for CDDP with NC (red) and WT (yellow) as reference.

To investigate the association between TP53 mutations and platinum resistance in OC, we profiled TP53 mutations in 96 high-grade serous ovarian cancer (HGSOC) tumor samples, stratified into four groups: primary sensitive (PS), primary resistant (PR), relapsed sensitive (RS), and relapsed resistant (RR) (Fig. 1A; Details in “Methods”). Somatic TP53 mutations were identified in 83.3% of patients, which mirrors the mutation prevalence reported in Asian populations (Fig. 1B) [6, 7]. Among these, missense mutations were predominant (57%) and were localized within the DBD (Supplementary Fig. S1A, B). The identified TP53 hotspot mutations, R175, Y220, R248, R273, and G245, were consistent with the most frequently mutated TP53 residues reported in both The Cancer Genome Atlas (TCGA) and Chinese patient cohorts (Fig. 1C–E).

Previous studies have predominantly focused on DBD hotspot mutations; however, the contribution of missense mutations in secondary or tertiary domains to platinum resistance remains poorly defined. Here, we comprehensively analyzed the distribution of missense mutations within the DBD across the four groups (Fig. 1F) and quantified the proportion of mutations within each secondary domain (Fig. 1G). Notably, mutations in Loop 3 (particularly amino acids 240–250) were significantly more prevalent in the PR group. Likewise, mutations in the β-strand S10 and Loop 2 were more frequent in the RR group by Fisher’s exact test (Tables S1–S3). Additionally, three-dimensional structural analyses revealed that mutations in the PR group predominantly clustered at the direct DNA-contact interface of the p53 protein (Supplementary Fig. S1C).

To validate the findings, representative mutations from the PR and RR groups were introduced into p53-null SKOV3 cells (Supplementary Fig. S1D–F; Table S4). Cell Counting Kit-8 (CCK-8) assay showed that these mutations significantly enhanced cellular tolerance to cisplatin (Fig. 1H, I), as reflected by elevated cisplatin IC_50_ values compared to the negative control (NC: 1.54 µM) and wild-type control (WT: 2.62 µM) (Fig. 1J, K; Table S5–S8). These statistical and functional assays indicated a close association between missense mutations in the Loop 2, Loop 3, and β-strand S10 regions of TP53 and platinum resistance in SKOV3 cells.

Notably, two relapse-resistant variants at Arg175 (R175H and R175G) exhibited distinct levels of cisplatin resistance, with R175G conferring nearly double the IC_50_ of R175H. Analysis of the National Cancer Institute (NCI) database revealed that R175H was more prevalent in colorectal cancer (16%), while R175G predominated in lung cancer (33%) (Supplementary Fig. S1G, H). Although both variants are linked to drug resistance, whether they share regulatory networks or possess unique functional roles warrants further investigation.

### p53^R175G^ confers enhanced cisplatin resistance and promotes tumor cell migration compared to p53^R175H^

**Fig. 2:**
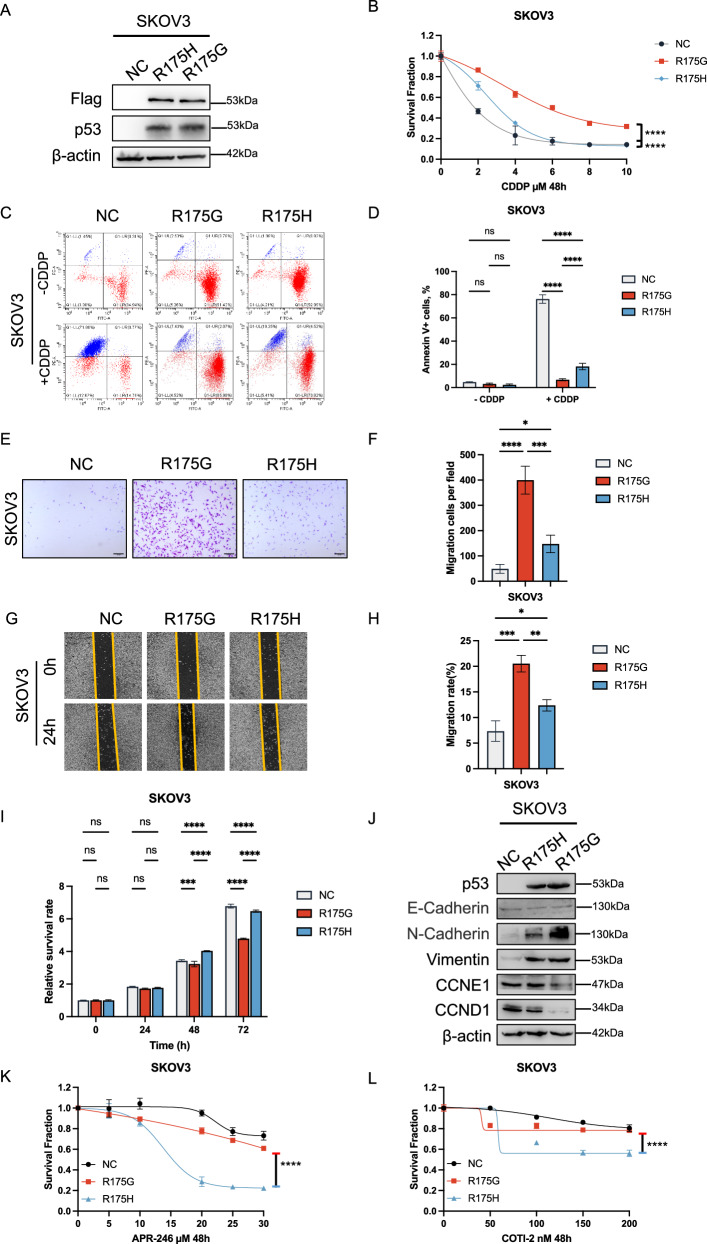
p53^R175G^ exhibits enhanced cisplatin resistance and cell migration promotion compared to p53^R175H^. **A** Western blot (WB) assay validating the successful establishment of p53-null (p53^NC^), p53^R175H^, and p53^R175G^ mutations in TP53-null SKOV3 cells. **B** CCK-8 assay validating the cisplatin resistance of p53^NC^, p53^R175G^ and p53^R175H^ in SKOV3 cells. The data are presented as a nonlinear fit. **C**, **D** Apoptosis assay validating the cisplatin-induced apoptosis of p53^NC^, p53^R175G^ and p53^R175H^ in SKOV3 cells. **E**, **F** Cell migration assay validating the cell migration capacity of p53^R175G^ and p53^R175H^ in SKOV3 cells. **G**, **H** Wound healing assay validating the wound healing rate of p53^NC^, p53^R175G^ and p53^R175H^ in SKOV3 cells. **I** CCK-8 assay validating the proliferation of p53^NC^, p53^R175G^ and p53^R175H^ in SKOV3 cells. **J** WB assay validating the expression levels of E-Cadherin, N-Cadherin, Vimentin, cyclin E1 (CCNE1) and cyclin D1 (CCND1) of p53^NC^, p53^R175G^ and p53^R175H^ in SKOV3 cells. **K**, **L** CCK-8 assay validating the sensitivity of p53^NC^, p53^R175G^ and p53^R175H^ to p53^R175H^-targeting drugs APR-246 and COTI-2. The data are presented as mean ± standard deviation: *P < 0.05, **P < 0.01, ***P < 0.001, ****P < 0.0001, significant difference; ns, no significant difference.

To further elucidate the functional differences between p53^R175G^ and p53^R175H^, we stably expressed p53^NC^ (negative control), p53^R175H^, and p53^R175G^ in p53-null SKOV3 and H1299 cells as well as in OVCAR8 cells that express endogenous p53 (Fig. 2A; Supplementary Figs. S2A and S3A). CCK-8 assay showed that both p53^R175G^ and p53^R175H^ significantly increased the resistance of tumor cells to cisplatin compared to the negative control, with p53^R175G^ conferring a markedly higher level of resistance than did p53^R175H^ (Fig. 2B; Supplementary Figs. S2B and S3B). Apoptosis assay further demonstrated that both variants significantly reduced cisplatin-induced apoptosis, with p53^R175G^ exhibiting the strongest anti-apoptotic effect (Fig. 2C, D; Supplementary Figs. S2C, D and S3C, D). Furthermore, cell migration assay revealed that both p53^R175G^ and p53^R175H^ substantially promoted the migratory potential of tumor cells relative to the negative control, with the pro-migratory effect of p53^R175G^ being particularly pronounced (Fig. 2E, F; Supplementary Figs. S2E, F and S3E, F). Wound healing assay corroborated these observations, further highlighting the superior migration-promoting effect of p53^R175G^ (Fig. 2G, H; Supplementary Figs. S2G, H and S3G, H). Notably, p53^R175G^ did not enhance the proliferation capacity of tumor cells; in fact, its proliferation rate was lower than that observed with p53^R175H^ (Fig. 2I; Supplementary Figs. S2I and S3I). To further compare the phenotypic characteristics of p53^R175G^ and p53^R175H^, we measured the expression of proliferation markers (cyclin E1, cyclin D1) and epithelial–mesenchymal transition markers (E-cadherin, N-cadherin, vimentin). Cells expressing p53^R175G^ exhibited elevated N-cadherin and vimentin levels relative to those expressing p53^R175H^, consistent with enhanced migratory capacity, whereas cyclin E1 expression was lower, indicating reduced proliferative potential (Fig. 2J; Supplementary Figs. S2J and S3J). Additionally, we assessed the sensitivity of p53^R175G^ to p53^R175H^-targeting drugs (APR-246, COTI-2) via CCK-8 assay. At concentrations that did not significantly affect p53^NC^ or p53^shTP53^cell viability, inhibition of p53^R175G^ cell viability was markedly less than that observed in p53^R175H^ cells (Fig. 2K, L; Supplementary Figs. S2K, L and S3K, L).

Taken together, these findings suggest that p53^R175G^ and p53^R175H^ possess distinct functional characteristics, with p53^R175G^ exhibiting stronger cisplatin resistance and migration-promoting properties than p53^R175H^. These findings indicate that the two variants may function through different molecular mechanisms.

### Distinct transcriptomic signatures underlying cisplatin resistance in p53^R175G^ and p53^R175H^

**Fig. 3:**
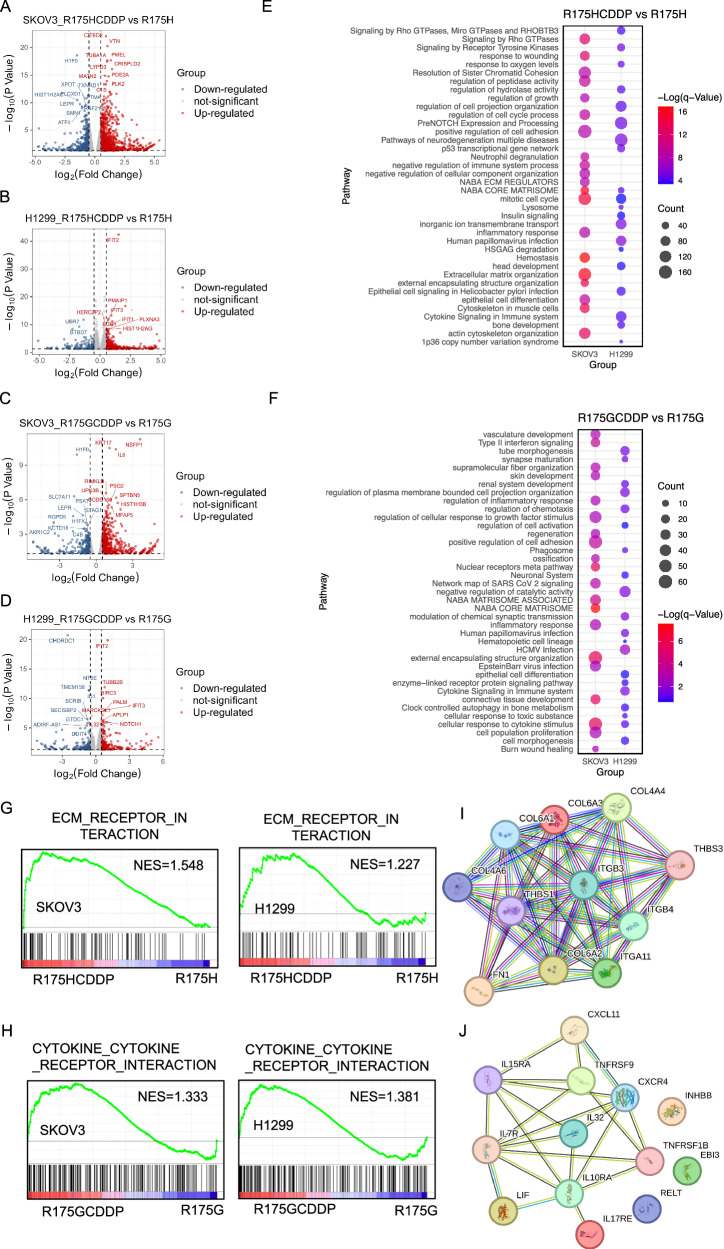
Transcriptomic analysis reveals key drivers of cisplatin resistance in p53^R175G^ and p53^R175H^ cells. **A**, **B** Volcano plots for DEGs in p53^R175H^ cells treated with cisplatin, analyzed in SKOV3 and H1299 cells. Colored dots represented DEGs with *p* < 0.05 and fold change ≥1.5. **C**, **D** Volcano plots for DEGs in p53^R175G^ cells treated with cisplatin, analyzed in SKOV3 and H1299 cells. Colored dots represented DEGs with *p* < 0.05 and fold change ≥1.5. **E** Enriched pathways of upregulated DEGs in R175HCDDP compared to R175H were identified using Metascape, and *p* values calculated via a one-sided Fisher’s exact test. **F** Enriched pathways of upregulated DEGs in R175GCDDP compared to R175G were identified using Metascape, and *p* values calculated via a one-sided Fisher’s exact test. **G**, **H** GSEA enrichment plot for DEGs in p53^R175H^ and p53^R175G^ cells treated with cisplatin. Nominal *p* < 0.05. **I** Key factors involved in the ECM receptor interaction pathway. **J** Key factors involved in the cytokine-cytokine receptor interaction pathway.

To further delineate the cofactors and regulatory networks associated with p53^R175H^ and p53^R175G^ in promoting tumor progression, RNA sequencing (RNA-seq) was conducted on SKOV3 and H1299 cell lines stably expressing either p53^R175H^ or p53^R175G^, before and after cisplatin treatment. Differentially expressed genes (DEGs) were identified, and enrichment analysis prioritized genes and pathways that were consistently regulated across both cell lines, enhancing the robustness of the findings. Volcano plots illustrated genes associated with p53^R175H^ that were significantly upregulated, downregulated, or unchanged following cisplatin treatment (Fig. 3A, B). Significantly upregulated genes were further analyzed using Metascape, revealing enrichment of pathways such as the NABA CORE MATRISOME, mitotic cell cycle, and signaling by Rho GTPases in both SKOV3 and H1299 cells treated with cisplatin (R175HCDDP group, Fig. 3E). Additionally, gene set enrichment analysis (GSEA) demonstrated a positive correlation between the ECM-receptor interaction pathway and the R175HCDDP group, involving core regulatory molecules such as *COL4A6*, *COL6A1*, *FN1*, *ITGB3*, and *THBS1* (Fig. 3G, I). A parallel approach was used to examine the transcriptomic response of p53^R175G^ cells to cisplatin (Fig. 3C, D). Functional enrichment analyses indicated significant activation of the cellular response to cytokine stimulus pathway in both cell lines (Fig. 3F). Moreover, GSEA revealed a positive correlation between the cytokine-cytokine receptor interaction pathway and the R175GCDDP group, with key molecules including *IL7R*, *IL32*, *TNFRSF9*, *CXCL11*, *CXCR4*, and *INHBB* (Fig. 3H, J).

**Fig. 4:**
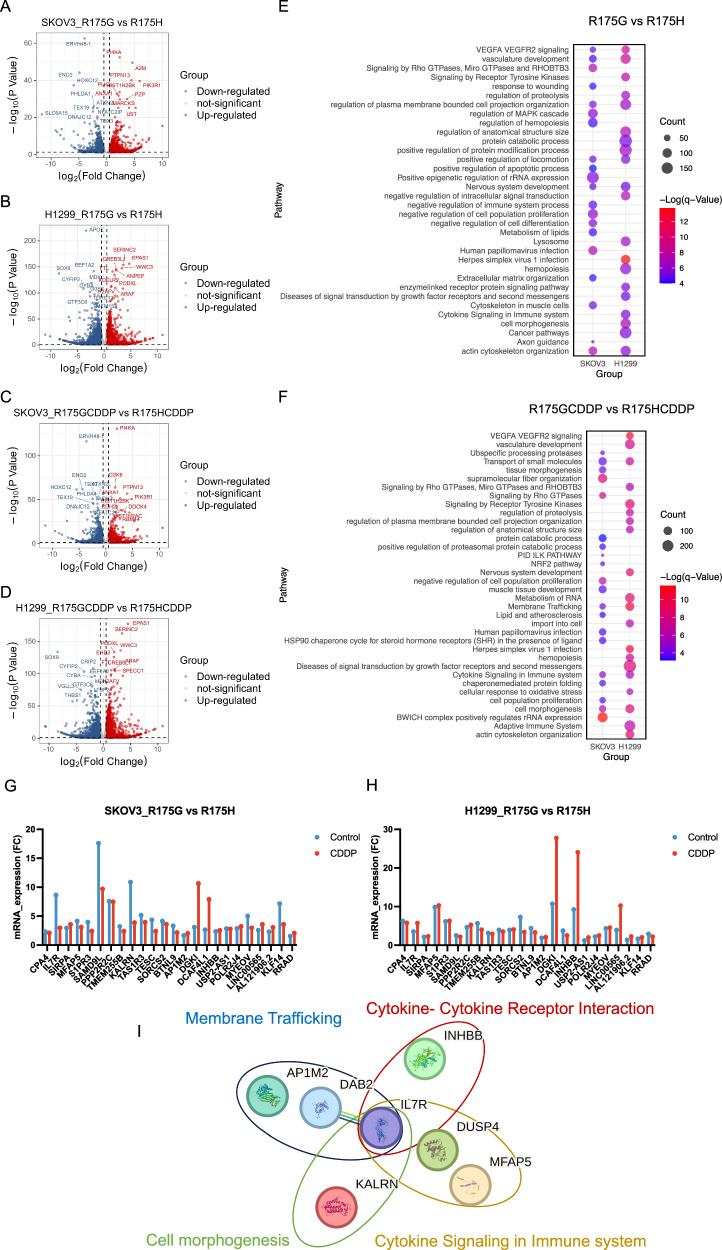
Differential pathway activation in p53^R175G^ and p53^R175H^ cells: implications for cisplatin resistance and cell migration. **A**–**D** Volcano plots for the DEGs between p53^R175G^ and p53^R175H^ in SKOV3 and H1299 cells before and after cisplatin treatment. Colored dots indicated DEGs with *p* < 0.05 and fold change ≥1.5. **E** Enriched pathways of upregulated DEGs in R175G compared to R175H were identified using Metascape, with *q* values calculated using a one-sided Fisher’s exact test. **F** Enriched pathways of upregulated DEGs in R175GCDDP compared to R175HCDDP were also identified using Metascape, with *q* values calculated using a one-sided Fisher’s exact test. **G**, **H** Significant upregulated DEGs in R175GCDDP compared to R175HCDDP in SKOV3 and H1299 cells (fold change ≥ 2, padj < 0.05). **I** *IL7R* is implicated in cytokine-cytokine receptor interaction, membrane trafficking, cytokine signaling in immune system, and cell morphogenesis pathways.

To further dissect the molecular distinctions between p53^R175G^ and p53^R175H^ in driving cancer progression, genes that were significantly upregulated in p53^R175G^ compared with p53^R175H^ (Fig. 4A, B) were subjected to functional enrichment analysis. The analysis revealed abnormal activation of VEGFA-VEGFR2 signaling and vasculature development pathways in the p53^R175G^ group, which may underlie its stronger pro-migration capacity (Fig. 4E). Membrane trafficking, cytokine signaling in immune system, and cell morphogenesis pathways were also enriched in the p53^R175GCDDP^ group, further suggesting an association with cisplatin resistance (Fig. 4C, D, F). To identify key drivers of p53^R175G^-mediated cisplatin resistance, we identified DEGs between R175GCDDP and R175HCDDP following cisplatin treatment (fold change ≥ 2, padj < 0.05) and filtered for those shared by both SKOV3 and H1299 cells, resulting in 24 candidate genes (Supplementary Fig. S4A–C). Among these, interleukin-7 receptor (*IL7R*) was markedly overexpressed in p53^R175G^ (Fig. 4G, H). Functional enrichment revealed that *IL7R* was involved in cytokine-cytokine receptor interactions, membrane trafficking, cytokine signaling in immune system, and cell morphogenesis pathways, suggesting that *IL7R* may drive the oncogenic phenotype of p53^R175G^ through multidimensional regulation (Fig. 4I). In summary, these analyses demonstrate that p53^R175G^ and p53^R175H^ mediate cisplatin resistance and cell migration via distinct molecular mechanisms: p53^R175G^-driven resistance is associated with upregulation of *IL7R* and activation of cytokine-related pathways, whereas p53^R175H^ relies more on ECM-related and cell cycle regulatory networks.

### Pharmacological targeting of IL7R overcomes p53^R175G^-driven tumor progression and enhances cisplatin efficacy

**Fig. 5:**
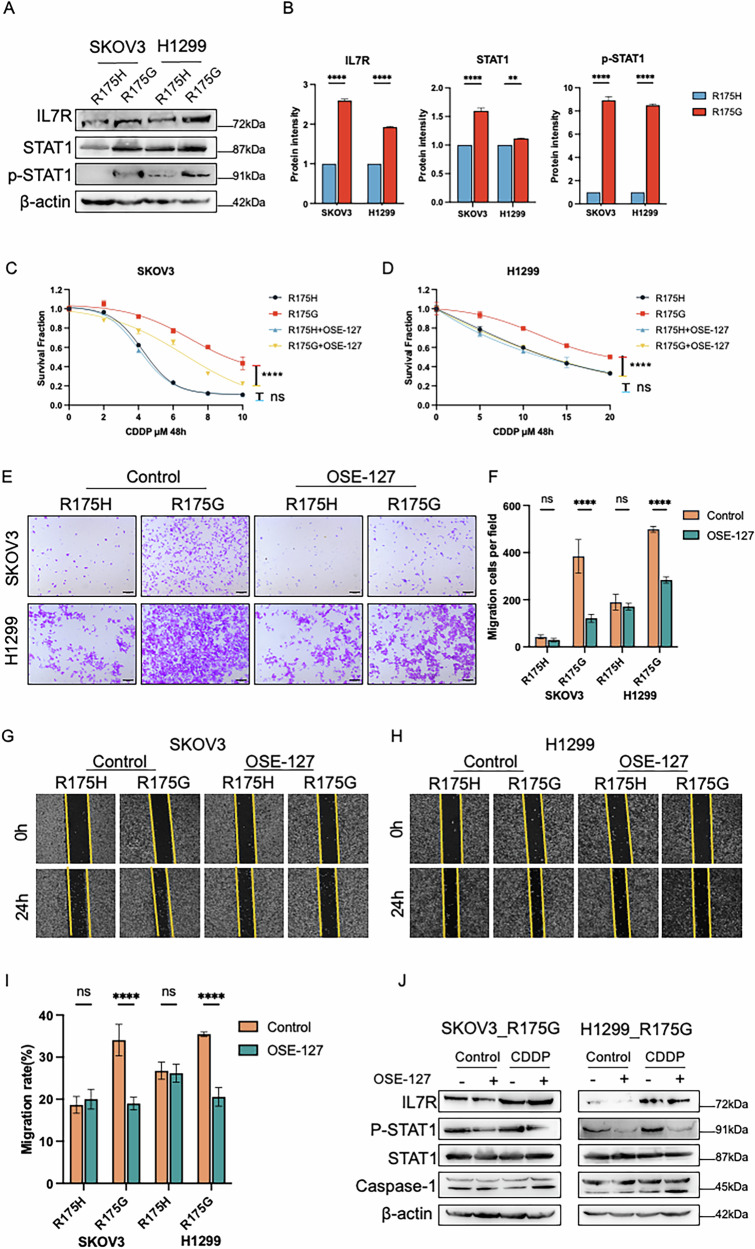
Targeting IL7R with OSE-127 restores cisplatin sensitivity and inhibits cell migration in p53^R175G^ cells. **A** WB assay validating the expression levels of IL7R, STAT1, and phosphorylated STAT1 (p-STAT1) in p53^R175G^ and p53^R175H^ cells. **B** Expression validation of IL7R, STAT1, and p-STAT1 in SKOV3 and H1299 cells accompanied by the protein band intensities relative to β-Actin in scrambled cells. **C**, **D** CCK-8 assay validating that OSE-127 (10 ng/ml) significantly improved the cisplatin sensitivity of p53^R175G^ cells. **E**–**I** Cell migration and wound healing assays validating that OSE-127 (10 ng/ml) significantly suppressed the migration of p53^R175G^ in both SKOV3 and H1299 cell lines. **J** WB assay validating the expression levels of IL7R, STAT1, p-STAT1, and Caspase-1 in p53^R175G^ cells treated with cisplatin alone, OSE-127 (10 ng/ml) alone, or the combination of both. The data are presented as mean ± standard deviation: ****P < 0.0001, significant difference; ns, no significant difference.

Previous studies have shown that IL7R promotes tumor cell survival by activating the JAK-STAT pathway [37]. To further explore the biological functions of IL7R in p53^R175G^ cells, we first assessed IL7R expression using WB assay. The results revealed that IL7R protein levels were significantly higher in p53^R175G^ cells compared to p53^R175H^ cells, and this elevation was accompanied by a marked increase in STAT1 phosphorylation (p-STAT1) (Fig. 5A, B). To further substantiate the synergistic effects of OSE-127 and cisplatin, we evaluated the pharmacological inhibition of IL7R by OSE-127 in combination with cisplatin in p53^R175G^ cells by CCK-8 assay. The combination exhibited synergistic effects, as demonstrated by the Chou–Talalay method in both cell lines (Supplementary Fig. S4D–G), whereas no significant effect was observed in p53^R175H^ cells (Fig. 5C, D). Similarly, cell migration and wound healing assays indicated that OSE-127 treatment markedly inhibited migration capacity of p53^R175G^ cells but had no significant effect on p53^R175H^-mediated migration (Fig. 5E–I). WB assay further showed that OSE-127 treatment alone significantly reduced p-STAT1 in p53^R175G^ cells, while cisplatin treatment alone increased p-STAT1. Moreover, combined OSE-127 and cisplatin treatment reversed p-STAT1 activation and upregulated the expression of the pro-apoptotic protein Caspase-1. Under cisplatin stress, OSE-127 effectively inhibited the p-STAT1 downstream of IL7R in p53^R175G^ cells, even when IL7R expression was increased (Fig. 5J). These findings indicate that high IL7R expression promotes p-STAT1 activation, driving cisplatin resistance and migration in p53^R175G^ cells, while IL7R pharmacological inhibition markedly suppresses tumor progression and enhances the efficacy of cisplatin treatment.

### Distinct and shared transcriptional regulation of p53^R175G^ and p53^R175H^

p53 is a key transcription factor, and its mutant forms can regulate the genome by interacting with other transcription factors, mediating chromatin remodeling and acquiring specific DNA-binding capabilities [38, 39]. To explore the transcriptional regulatory roles of p53^R175H^ and p53^R175G^, we performed chromatin immunoprecipitation sequencing (ChIP-seq) on SKOV3 and H1299 cell lines. p53^R175G^ and p53^R175H^ displayed both shared and distinct genome-binding profiles, with SKOV3 cells showing 6092 overlapping peaks and H1299 cells 902. Furthermore, read distribution and peak localization differed between the variants (Supplementary Fig. S5A–D). These results indicated that while p53^R175G^ and p53^R175H^ co-regulate some targets, each mutant also binds unique genomic regions.

**Fig. 6:**
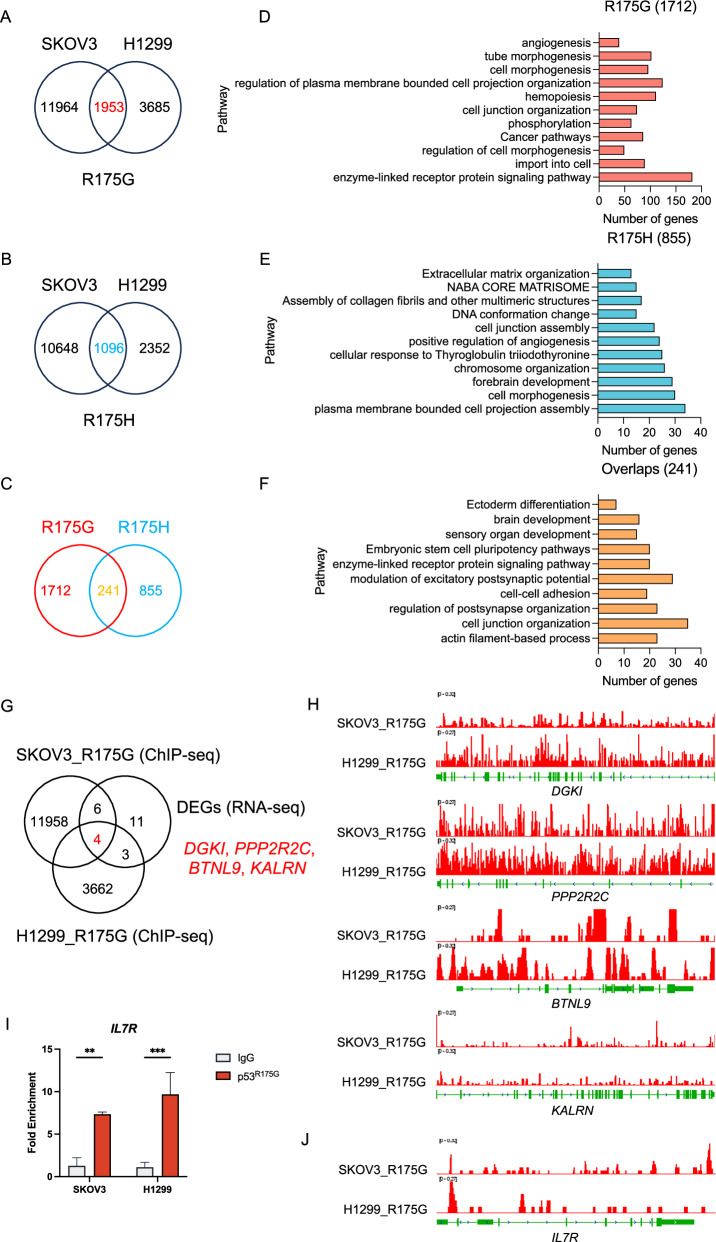
Comparative analysis of transcriptional regulation by p53^R175G^ and p53^R175H^ via ChIP-seq and RNA-seq. **A** Wayne diagram showing p53^R175G^ binding genes in SKOV3 and H1299 cells. **B** Wayne diagram showing p53^R175H^ binding genes in SKOV3 and H1299 cells. **C** Wayne diagram showing peak-associated genes targeted by p53^R175G^ and p53^R175H^. **D** Pathway enrichment plot for genes in p53^R175G^-target (1 712 genes). **E** Pathway enrichment plot for genes in p53^R175H^-target (855 genes). **F** Pathway enrichment plot for genes jointly bound by p53^R175G^ and p53^R175H^ (241 genes). **G** Wayne diagram showing p53^R175G^-target genes (ChIP-seq) and DEGs (fold change ≥ 2, padj < 0.05) from RNA-seq (R175GCDDP vs R175HCDDP). **H** ChIP-seq signals for p53^R175G^ target genes, including *DGKI*, *PPP2R2C*, *BTNL9* and *KALRN*. **I** ChIP-qPCR assay showing p53^R175G^ enrichment at the promoter region of *IL7R*. **J** ChIP-seq signal showing p53^R175G^ binding to the *IL7R* gene.

In SKOV3 and H1299 cells, 241 peak-associated genes were co-bound by p53^R175G^ and p53^R175H^, while 1712 and 855 genes were uniquely associated with p53^R175G^ and p53^R175H^, respectively (Fig. 6A–C). Functional enrichment analysis revealed that p53^R175G^-bound genes were mainly involved in signaling, phosphorylation, morphogenesis, and angiogenesis (Fig. 6D), whereas p53^R175H^-bound genes were enriched in extracellular matrix organization, chromosome organization, positive regulation of angiogenesis, and related pathways (Fig. 6E). Co-bound genes were linked to actin filament processes, cell junction organization, and cell-cell adhesion (Fig. 6F). Integration of RNA-seq and ChIP-seq analyses further demonstrated that while p53^R175H^ and p53^R175G^ co-regulate angiogenesis-related genes, they promote tumor progression via distinct pathways: p53^R175H^ upregulates extracellular matrix genes, while p53^R175G^ activates membrane trafficking and cell morphogenesis pathways.

To further identify the direct transcriptional targets of p53^R175G^, we overlapped the p53^R175G^-bound genes with the 24 DEGs identified in the RNA-seq comparison between R175GCDDP and R175HCDDP. 13 direct transcriptional targets were identified, including *DGKI*, *PPP2R2C*, *KALRN*, and *BTNL9*, which were bound by p53^R175G^ in both SKOV3 and H1299 cells (Fig. 6G). ChIP-seq data confirmed that p53^R175G^ bound to the transcription start site (TSS) regions of these four genes (Fig. 6H). Chromatin immunoprecipitation-quantitative (ChIP)-qPCR assay was conducted and revealed that p53^R175G^ was enriched approximately sevenfold at the *IL7R* promoter compared to the IgG negative control (Fig. 6I), consistent with ChIP-seq signal showing a binding peak in the *IL7R* promoter region (Fig. 6J). Together, these results indicated that while both p53^R175G^ and p53^R175H^ co-regulate angiogenesis-related genes, p53^R175G^ upregulates *IL7R* and other oncogenic targets, whereas p53^R175H^ upregulates extracellular matrix-related genes.

### CHD1 specifically interacts with p53^R175G^

**Fig. 7:**
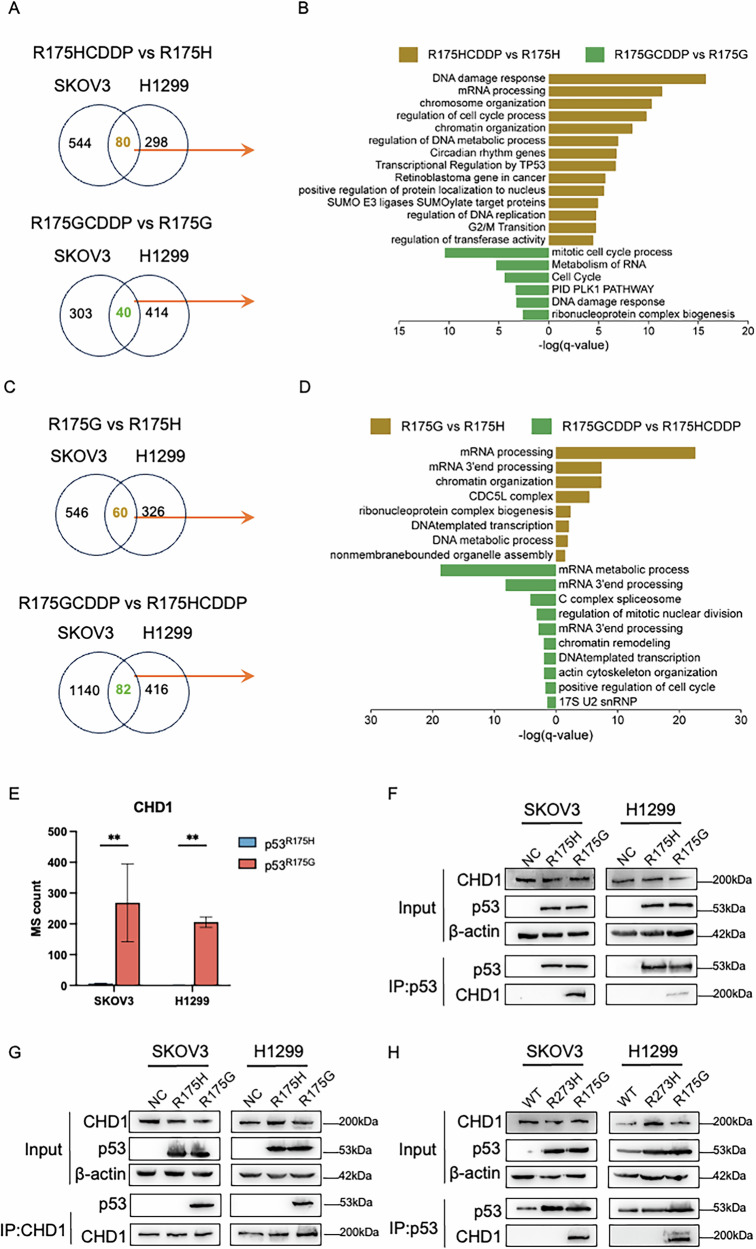
Identification of the interacting proteomes of p53^R175H^ and p53^R175G^ was performed using co-immunoprecipitation-mass spectrometry (IP-MS). **A** Wayne diagram showing the differentially expressed proteins (DEPs) of p53^R175H^ or p53^R175G^ before and after cisplatin treatment in SKOV3 and H1299 cells. DEPs were defined as proteins with a *p* < 0.05 and a fold change ≥1.5. **B** Pathway enrichment plot for the overlapping DEPs (in **A**) was conducted using Metascape, with *q* values calculated via a one-sided Fisher’s exact test. **C** Wayne diagram showing the DEPs (R175G vs R175H) before and after cisplatin treatment in SKOV3 and H1299 cells. DEPs were defined as proteins with a *p* < 0.05 and a fold change ≥1.5. **D** Pathway enrichment plot for the overlapping DEPs (in **C**) was conducted using Metascape, with *q* values calculated via a one-sided Fisher’s exact test. **E** CHD1 was identified as a key interacting protein specific to p53^R175G^ in SKOV3 and H1299 cells. **F**, **G** Reciprocal Co-IP of CHD1 with p53^NC^, p53^R175G^ and p53^R175H^ in SKOV3 and H1299 cells. **H** Co-IP of CHD1 with p53^WT^, p53^R273H^ and p53^R175G^ in SKOV3 and H1299 cells.

To investigate the underlying mechanism by which p53^R175H^ and p53^R175G^ promote tumor progression, co-immunoprecipitation-mass spectrometry (IP-MS) was applied to characterize their interacting protein profiles. Several proteins previously reported as p53^R175H^ interactors, such as TP53BP1, USP28, and SP1, were identified. Both variants were found to interact with proteins involved in cell cycle regulation and DNA damage repair (Fig. 7A, B). Notably, pathway analysis revealed that p53^R175G^, compared to p53^R175H^, exhibited significant activation of pathways related to mRNA processing, cell cycle progression, and chromatin remodeling, particularly following cisplatin exposure (Fig. 7C, D).

Among the chromatin remodeling proteins, Chromodomain-helicase-DNA-binding protein 1 (CHD1) exhibited a notably stronger interaction with p53^R175G^ than with p53^R175H^ (Fig. 7E). This preferential interaction was further validated by reciprocal co-immunoprecipitation (Co-IP) assays. In these experiments, CHD1 was immunoprecipitated and probed for p53 to confirm the bidirectional nature of the interaction (Fig. 7F, G). To assess the specificity of this interaction, we examined the binding of CHD1 to p53^WT^ and p53^R273H^ in SKOV3 and H1299 cells. Neither p53^WT^ nor p53^R273H^ showed detectable interaction with CHD1, in contrast to the strong association observed with p53^R175G^ (Fig. 7H), thereby highlighting the specificity of the R175G–CHD1 axis. Furthermore, survival analysis demonstrated that high CHD1 expression correlated with poorer prognosis in patients with p53-mutant OC (log-rank *P* < 0.05) (Supplementary Fig. S5E).

**Fig. 8:**
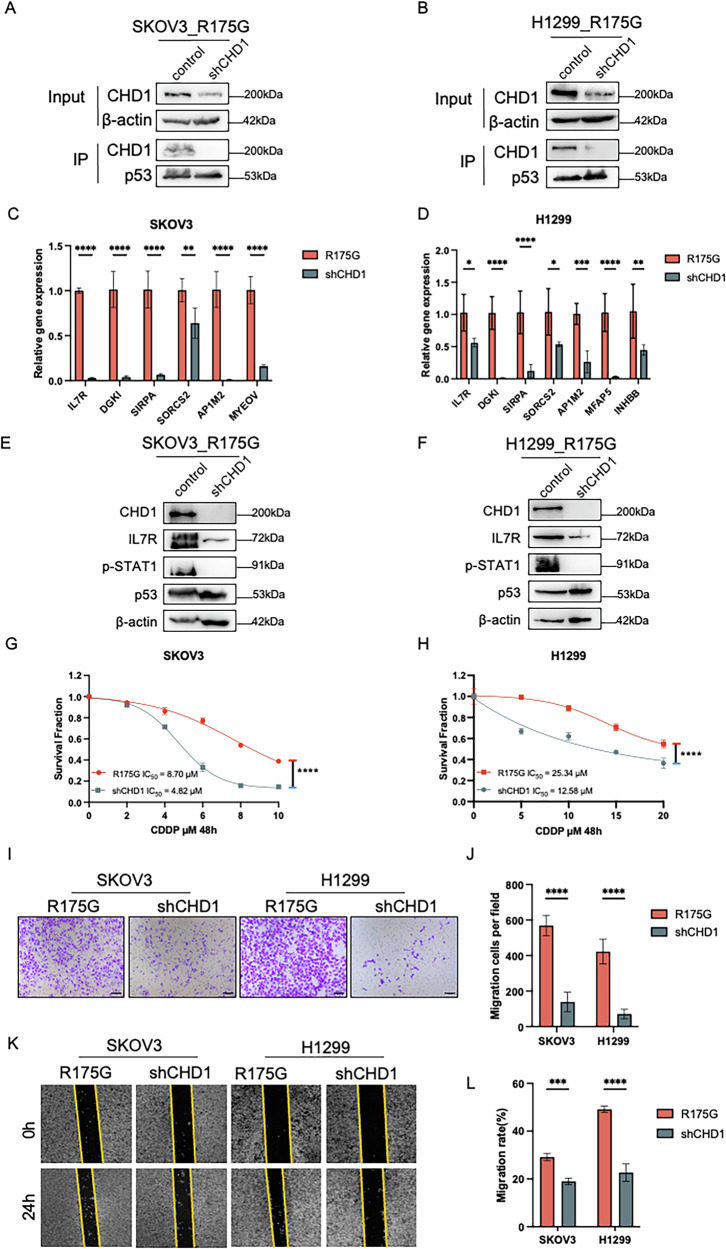
Roles of CHD1 in regulating p53^R175G^-driven gene expression and tumor progression. **A**, **B** WB assay demonstrated that CHD1 knockdown using siRNA disrupted its interaction with p53^R175G^. **C** RT-PCR assay showed that CHD1 knockdown significantly downregulated the mRNA levels of p53^R175G^-driven genes, including *IL7R*, *DGKI*, *SIRPA*, *SORCS2*, *AP1M2*, and *MYEOV* in SKOV3 cells. **D** RT-PCR assay showed that CHD1 knockdown significantly decreased the mRNA levels of p53^R175G^-driven genes, including *IL7R*, *DGKI*, *DCAF4L1*, *SIRPA*, *SORCS2*, *AP1M2*, *MFAP5*, and *INHBB* in H1299 cells. **E**, **F** WB assay showed that CHD1 knockdown decreased IL7R protein levels and reduced p-STAT1 expression in SKOV3 and H1299 cells. **G**, **H** CCK-8 assay showed that CHD1 knockdown significantly reduced cisplatin resistance of p53^R175G^ in SKOV3 and H1299 cells. **I**, **J** Cell migration assay showed that CHD1 knockdown inhibited the migration ability of p53^R175G^ in SKOV3 and H1299 cells. **K**, **L** Wound healing assay showed that CHD1 knockdown significantly reduced the wound healing rate of p53^R175G^ in SKOV3 and H1299 cells. The data are presented as mean ± standard deviation: *P < 0.05, ***P < 0.001, and ****P < 0.0001, significant differences.

To further explore the functional significance of CHD1, we knocked down CHD1 by siRNA and observed a concomitant reduction in its association with p53^R175G^ cells (Fig. 8A, B). CHD1 silencing also significantly decreased the expression of multiple p53^R175G^ target genes, including *IL7R*, *DGKI*, *SIRPA*, *MYEOV*, and *INHBB* (Fig. 8C, D and Supplementary Fig. S5F), using RT-qPCR analyses. A luciferase assay targeting the *IL7R* promoter demonstrated that p53^R175G^ overexpression increased *IL7R* activity in HEK293T cells, and this effect was significantly suppressed by CHD1 depletion (Supplementary Fig. S5G). Moreover, ablation of CHD1 by siRNA dramatically prevented the upregulation of IL7R and p-STAT1 in p53^R175G^ cells (Fig. 8E, F). Functional assays revealed that CHD1 knockdown, in combination with cisplatin treatment, significantly enhanced tumor suppression and reduced the cisplatin IC_50_ by ~50% compared to cisplatin alone in p53^R175G^ cells (Fig. 8G, H). Additionally, cell migration and wound healing assays showed that CHD1 silencing markedly impaired the migratory capacity of p53^R175G^ cells (Fig. 8I–L). Collectively, these findings demonstrate that CHD1 supports p53^R175G^-driven transcriptional activation of oncogenic genes such as *IL7R*, promoting tumor progression. Conversely, depletion of CHD1 synergistically sensitizes p53^R175G^ cells to cisplatin and suppresses migratory potential, providing a rationale for combination therapies specifically targeting the R175G mutation.

## Discussion

Although the functional heterogeneity of TP53 mutations is recognized [26–31, 40], the functional differences and mechanistic underpinnings of TP53 mutations at the same amino acid remain poorly understood. In this study, we have demonstrated that p53^R175H^ and p53^R175G^ exerted distinct effects on cell migration and platinum resistance. Remarkably, even substitutions at the same codon could exhibit unique cofactor interactions. For instance, p53^R175G^, but not ^p53R175H^, specifically interacted with CHD1. This unique interaction regulated the transcriptional activity of p53^R175G^ and modulated the IL7R-STAT1 signaling pathway, thereby promoting tumor progression. Furthermore, inhibition of either CHD1 or IL7R sensitized p53^R175G^ cells to platinum, suggesting a potential therapeutic strategy for tumors harboring the R175G mutation.

Growing evidence demonstrates significant functional divergence among TP53 mutations at the same residue. For example, in non-small-cell lung cancer, R248Q mutation enhanced tumor invasiveness, while R248W did not [35]. Similarly, R273H and R273C mutations promoted proliferation, invasion, and drug resistance, while R273G mutation lacked these effects [36]. Recent studies on R175 variants indicated that R175H mutation primarily drove migration, invasion, and metastasis, whereas R175T and R175S mutations promoted apoptosis and cell cycle arrest, respectively [11]. In our study, we found that R175G mutation exerted a stronger effect on apoptosis compared to proliferation and exhibited superior platinum resistance and pro-migratory capacity compared to R175H mutation. Moreover, R175G mutation was unresponsive to targeted agents such as APR-246 and COTI-2, corroborating previous reports of resistance in R175 mutations [11]. These results underscore the importance of considering the functional heterogeneity and cofactor dependence of TP53 mutations in precision medicine, as both the molecular mechanisms and clinical intervention strategies may vary among mutations at the same residue, thus providing critical insights for personalized treatment approaches.

Our finding showed that CHD1 specifically interacted with p53^R175G^ to regulate a distinct transcriptional program, including the upregulation of IL7R, DGKI, SIRPA, MYEOV, and INHBB, which provided new insight into the epigenetic mechanisms of platinum resistance. As an ATP-dependent chromatin remodeling enzyme, CHD1 plays a crucial role in regulating chromatin structure, gene expression, DNA repair, and replication [41, 42]. Previous studies have indicated that CHD1 supports tumor growth by modulating metabolic genes such as HK2 and LDHA, with its inhibition suppressing proliferation and inducing apoptosis, particularly in PTEN-deficient models [43]. Extending these insights, our results suggested that CHD1 may regulate genes associated with platinum sensitivity and cell migration, making the development of selective CHD1 inhibitors or degraders a promising precision therapeutic strategy for R175G mutation cancers.

Similarly, cytokine signaling in promoting tumor progression and therapy resistance has been increasingly recognized [44–49]. IL7R activation is well documented in hematological malignancies such as acute lymphoblastic leukemia, where it drives pro-survival and proliferation pathways (notably via JAK/STAT, MAPK/ERK, and PI3K/AKT/mTOR signaling) [37, 50–54]. Our findings showed that IL7R was especially upregulated in p53^R175G^ cells, leading to enhanced STAT1 activation and extending the significance of IL7R signaling to mutant p53-driven solid tumors. While IL7/IL7R signaling was primarily associated with STAT5 activation, emerging reports and our findings indicated that STAT1 could also be engaged [55], imparting a dual function: although classically a tumor suppressor, STAT1 has been implicated in chemoresistance [56, 57] and immune evasion through induction of factors such as PD-L1 [58, 59] and ROS-scavenging proteins [60]. This aligns with our observation that STAT1 phosphorylation sustained platinum resistance and cell motility, while its inhibition sensitized cells to cisplatin-induced apoptosis. Taken together with the existing literature, these findings suggested that targeting CHD1 or IL7R may represent promising therapeutic strategies for overcoming platinum resistance and cell migration in p53^R175G^-mutant cancers.

Our findings on the distinct roles of p53^R175H^ and p53^R175G^ in cancer progression inspire promising research avenues. While structural biochemical analyses were not included, integrating crystallography and biophysical methods offers a prime opportunity to clarify how these mutations alter p53 structure/function and tumorigenic networks. Our discovery that CHD1/IL7R inhibition enhances platinum sensitivity in p53^R175G^ cells identifies novel therapeutic targets; future in vivo and clinical validation will strengthen translational potential and enable personalized strategies for p53-mutant cancers. Exploring other Arg175 mutations is critical, as these studies, combined with our results, will construct a comprehensive functional map of p53 mutations at Arg175.

In conclusion, our study elucidates the distinct functional roles and molecular mechanisms of p53^R175H^ and p53^R175G^ mutations in platinum-resistant OC. We demonstrate that mutations at the same arginine residue can drive tumor progression through divergent transcriptional programs and unique cofactor interactions, notably involving CHD1 in the case of p53^R175G^. These findings underscore the necessity of characterizing mutation-specific pathways and cofactor dependencies for the development of effective, individualized therapeutic strategies.

## Materials and methods

### Ethics approval and consent to participate

Secondary analyses of de-identified, publicly available data only. Original ethics approvals and informed consent for the HGSOC cohort were obtained by Qian et al. (Zhejiang Cancer Hospital IRB-2020-155; Westlake University 20190401GTN0009) in accordance with the Declaration of Helsinki. No new participants were enrolled and no identifiable information was used.

### Public data

TP53-targeted sequencing data from 96 HGSOC patients were obtained from the Genome Sequence Archive, accession number HRA007126 [2]. Patients were classified into four groups based on recurrence-free survival (RFS) following platinum chemotherapy: primary sensitive (PS, *n* = 27, RFS > 6 months), primary resistant (PR, *n* = 26, RFS ≤ 6 months), relapsed sensitive (RS, *n* = 26, RFS > 6 months), and relapsed resistant (RR, *n* = 17, RFS ≤ 6 months). Additional data on TP53 missense mutations were retrieved from TCGA, cBio Cancer Genomics Portal (cBioPortal), and NCI. All datasets are publicly available, and all analyses complied with respective data access and publication policies.

### Cell culture

All cell lines, including human OC cell lines (SKOV3, COV362, Kuramochi, OVCAR4, OVCAR8, A2780), human non-small cell lung cancer (H1299), and human embryonic kidney cells (293T), were all obtained from Wuhan Pricella Biotechnology Co., Ltd (Wuhan, China). Cells were cultured in Dulbecco’s Modified Eagle Medium (DMEM; Gibco, 11965092, Waltham, MA, USA) supplemented with 10% (v/v) fetal bovine serum (FBS; Excell, FSD500, Jiangyin, China) at 37 °C in a humidified incubator with 5% CO_2_.

### Lentiviral infection

Full-length TP53 was synthesized (Hangzhou Loche Biomedical Science and Technology Co., Ltd., Hangzhou, China), and mutations were introduced by site-directed mutagenesis using overlapping primers (Supplemental primers), then cloned into the pCDH-flagN-eGFP plasmid. The pLKO.1 plasmid was used for TP53 and CHD1 knockdown via siRNA.

Lentiviruses were produced in 293T cells by co-transfecting psPAX2, pMD2.G, and either pCDH-flagN-eGFP or pLKO.1 plasmids with TG transfection reagent. Viral supernatants were collected, filtered, and stored at −80 °C. For transduction, target cells were seeded in 24-well plates and infected with lentivirus and Polybrene (6 μg/ml; Beyotime, Shanghai, China). Medium was replaced after 24 h, and cells were cultured for an additional 48 h. Transfection efficiency was assessed by WB assay.

### Cell viability assay

The Cell Counting Kit-8 (CCK-8) assay was employed to evaluate cell viability. Cells were seeded into 96-well plates at a density of ~5000 cells per well and exposed to varying concentrations of cisplatin (Selleck, S1166, Houston, TX, USA), APR-246 (Selleck, S7724, Houston, TX, USA), COTI-2 (Selleck, S8580, Houston, TX, USA), or OSE-127 (MCE, HY-P99412, New Jersey, USA) for 48 h. Subsequently, 10 μl of CCK-8 reagent (Beyotime, Shanghai, China) was added to each well, followed by incubation at 37 °C for 1 h. The optical density at 450 nm was measured using a SpectraMax M5 Automated Microplate Reader (Molecular Devices, USA).

### Dose–response combination assays and calculation of drug combination index

For dose–response combination assays, cells were treated for 48 h with varying concentrations of two drugs, either alone or in combination. Cell viability was measured using a CCK-8 kit (Beyotime, Shanghai, China) according to the manufacturer’s protocol. Drug interactions were evaluated using CompuSyn software based on the Chou–Talalay method, which calculates the combination index (CI) via the isobologram equation, where CI < 1, =1, and >1 indicate synergism, additive effect, and antagonism, respectively [61].

### Cell migration assay

3 × 10⁴ cells were resuspended in 400 μl of serum-free medium and seeded into the upper chamber of a 24-well culture plate containing an 8.0 μm pore-size polycarbonate membrane (Corning, 3422, New York, USA). 1 ml of medium supplemented with 10% FBS was added to the lower chamber. After 24 h, the cells were fixed with 4% paraformaldehyde and stained with crystal violet (Beyotime, C0121, Shanghai, China) for 30 min. The migrated cells were imaged using an optical microscope.

### Wound healing assay

Cells were seeded into a 6-well plate and cultured until reaching 90% confluence the following day. A 200 μl pipette tip was used to create a scratch in the cell monolayer, simulating a wound. The medium was replaced with serum-free medium, and the cells were incubated for 24 h. Wound closure was imaged using a microscope (Olympus, Japan) and quantified with ImageJ software.

### Apoptosis assay

Due to the spontaneous green fluorescence of the cells, the Annexin V-PE apoptosis detection kit (Beyotime, C1065L, Shanghai, China), which emits red fluorescence, was selected to detect apoptosis signals. The procedure was conducted in accordance with the manufacturer’s instructions. Following cisplatin treatment for 48 h, both the supernatant and cells were collected. The cells were resuspended in 195 μl of Annexin V-PE binding solution, and 5 μl of Annexin V-PE was added. The mixture was incubated in the dark for 20 min and analyzed using flow cytometry (FACSCALBUR, BD Biosciences, USA).

### Co-immunoprecipitation (Co-IP) and Western blot assays

Cells were lysed in RIPA lysis buffer (Beyotime, P0013C) supplemented with 1% protease inhibitors. Anti-FLAG M2 beads (Sigma, M8823, Saint Louis, USA) were incubated with cell lysates overnight at 4 °C to immunoprecipitate FLAG-tagged proteins. The beads were washed, and the eluted proteins were separated by sodium dodecyl sulfate-polyacrylamide gel electrophoresis (SDS-PAGE). The proteins were transferred onto a polyvinylidene fluoride membrane (Millipore, IPFL00010, Massachusetts, USA). The membrane was blocked with 5% skim milk at room temperature for 1 h, followed by overnight incubation at 4 °C with primary antibodies. The following day, the membrane was incubated with horseradish peroxidase (HRP)-conjugated secondary antibodies (Abclonal, 1:10,000, Wuhan, China) at room temperature for 1 h. Protein bands were detected using enhanced chemiluminescence (ECL) reagents (Solarbio, Beijing, China).

The primary antibodies used were as follows: anti-FLAG (AE004, 1:4000; ABclonal, Wuhan, China), anti-β-actin (66009-1-Ig, 1:5000; Proteintech, Wuhan, China), IL7R (A11678, 1:2000; ABclonal, Wuhan, China), STAT1 (A19563, 1:2000; ABclonal, Wuhan, China), Phospho-STAT1-Y701 (AP0135, 1:2000; ABclonal, Wuhan, China), CHD1 (1:1000, Santa Cruz, Texas, USA), p53 (10442-1-AP, 1:10,000; Proteintech, Wuhan, China), HRP Goat Anti-Rabbit IgG (AS014, 1:10,000; ABclonal, Wuhan, China), HRP Goat Anti-Mouse IgG (AS003, 1:10,000; ABclonal, Wuhan, China), Caspase-1 (P79884R2S, 1:1000, Abmart, Shanghai, China). E-Cadherin (A20798, 1:1000; ABclonal, Wuhan, China), N-Cadherin (A19083, 1:1000; ABclonal, Wuhan, China), Vimentin (A19607, 1:1000; ABclonal, Wuhan, China), Cyclin E1 (11554-1-AP, 1:1000, Proteintech, Wuhan, China), and Cyclin D1 (60186-1-Ig, 1:1000, Proteintech, Wuhan, China).

### RNA extraction, RT-PCR and qRT-PCR analysis

Total RNA was extracted from treated cells using the NcmSpin Cell/Tissue Total RNA kit (NCM, M5105) following the manufacturer’s protocol. Complementary DNA (cDNA) was synthesized via reverse transcription (RT-PCR) using the PrimeScript RT reagent kit (Takara, RR037Q, Japan). Quantitative real-time PCR (qRT-PCR) was performed using the TB Green Premix Ex Taq II kit (Takara, RR820Q, Japan) on a Bio-Rad CFX Opus 96 Real-Time PCR System in accordance with the manufacturer’s instructions. The primers used are listed in the Supplemental Materials.

### Dual-luciferase reporter assay

To evaluate the effect of p53^R175G^ and CHD1 on *IL7R* promoter activity, the *IL7R* promoter, TP53 R175G, and siCHD1 sequences were cloned into the pGL4-Luc and pCDNA3.1-HA vectors, respectively, and co-transfected into HEK293T cells for 48 h. Luciferase activity was measured using the Dual-Luciferase Reporter Assay System (Promega, E1910), with firefly signals normalized to Renilla luciferase activity.

### RNA sequencing (RNA-seq)

RNA-seq was performed on SKOV3 and H1299 cell lines treated with or without 1 µM cisplatin (Selleck, S1166, Houston, TX, USA), with three biological replicates per group. Total RNA was extracted and assessed for quality using an Agilent 2100 Bioanalyzer. Libraries were prepared using the NEB general protocol, quantified with a Qubit 2.0 Fluorometer, and evaluated for insert size before sequencing on an Illumina platform. Library preparation, sequencing, and data analysis were conducted by Novogene Co., Ltd. (Beijing, China).

### Immunopurification–mass spectrometry (IP-MS)

SKOV3 and H1299 cells expressing FLAG-p53 were lysed in IP buffer (50 mM Tris-HCl, pH 7.5, 150 mM NaCl, 1% NP-40, and 1 mM EDTA) supplemented with a protease inhibitor cocktail (Selleck, B14001, Houston, TX, USA). The lysates were incubated overnight with Anti-FLAG M2 beads (Sigma, M8823, Saint Louis, USA) at 4 °C. The beads were washed, enzymatically digested with mass spectrometry-grade trypsin (Promega, V5280), desalted using Pierce C18 Tips (Thermo Scientific™, 87784, USA), vacuum-dried, and frozen. The resulting peptides were analyzed using Orbitrap Exploris™ 480 (Thermo Fisher Scientific, USA), and raw data were processed with MaxQuant software for protein identification and label-free quantification.

### Chromatin immunoprecipitation (ChIP) assay

Cells were crosslinked with 1% formaldehyde for 10 min at room temperature and quenched with 2.5 M glycine. After washing with cold TBS, cells were lysed in cell lysis buffer (10 mM Tris-HCl, pH 7.5, 10 mM NaCl, 0.5% NP-40) and incubated on ice for 10 min. For 3–4 × 10⁶ cells, chromatin was digested in 0.5 ml MNase digestion buffer with 5 µl of 1:10 diluted MNase (NEB, M0247S) at 37 °C with shaking for 20 min. Chromatin was sheared by sonication. 1% Input was retained at −20 °C. Immunoprecipitation was performed overnight at 4 °C with indicated antibodies, followed by incubation with protein A/G beads at 4 °C for 3 h. Beads were washed sequentially with 1× Stop/ChIP buffer, high-salt buffer, Tris/LiCl buffer, and TE buffer twice. Chromatin was eluted in elution buffer (10 mM Tris-HCl, pH 8.0, 10 mM EDTA, 150 mM NaCl, 5 mM DTT, 1% SDS) at 65 °C, and crosslinks were reversed by overnight incubation at 65 °C. RNA and proteins were removed by RNase A (TRAN, GE101-01, Beijing, China) and Proteinase K digestion (TRAN, GE201-01, Beijing, China), respectively. DNA was purified using a PCR Purification Kit (Qiagen, 28106, Hilden, Germany) and analyzed by qRT-PCR for target enrichment; primers are listed in the supplemental materials. ChIP-seq libraries were prepared and sequenced on an Illumina platform (Novogene Co., Ltd., Beijing, China).

### Statistical analysis

Each experiment in this study was conducted at least three times. Data analysis was performed using GraphPad Prism 10.0 and R 4.3.2. Statistical comparisons were evaluated using Fisher’s exact test, Student’s *t*-test, and one-way ANOVA. Statistical significance was indicated as follows: *P < 0.05, **P < 0.01, ***P < 0.001, and ****P < 0.0001.

## Supplementary information

Supplemental Figures and Figure legends

Supplementary Tables

original western blot data

## Data Availability

The raw RNA-seq and ChIP-seq data are deposited in the Sequence Read Archive (SRA) under accession ID PRJNA1233431 and PRJNA1245600. Additional data are available within the Article, and Supplementary Information.
