# Supplementary material for: Distinct functional heterogeneity of TP53 R175 mutations in platinum-resistant ovarian cancer: unveiling molecular mechanisms and therapeutic targets
Source: Cell Death Dis. 2025 Nov 17;16(1):837. doi: 10.1038/s41419-025-08172-0 (PMC12623820; doi:10.1038/s41419-025-08172-0)
Supplement: Supplementary file 1 — Supplemental Figures and Figure legends [file 41419_2025_8172_MOESM1_ESM.docx]

**Supplemental Figures and Figure Legends**

**
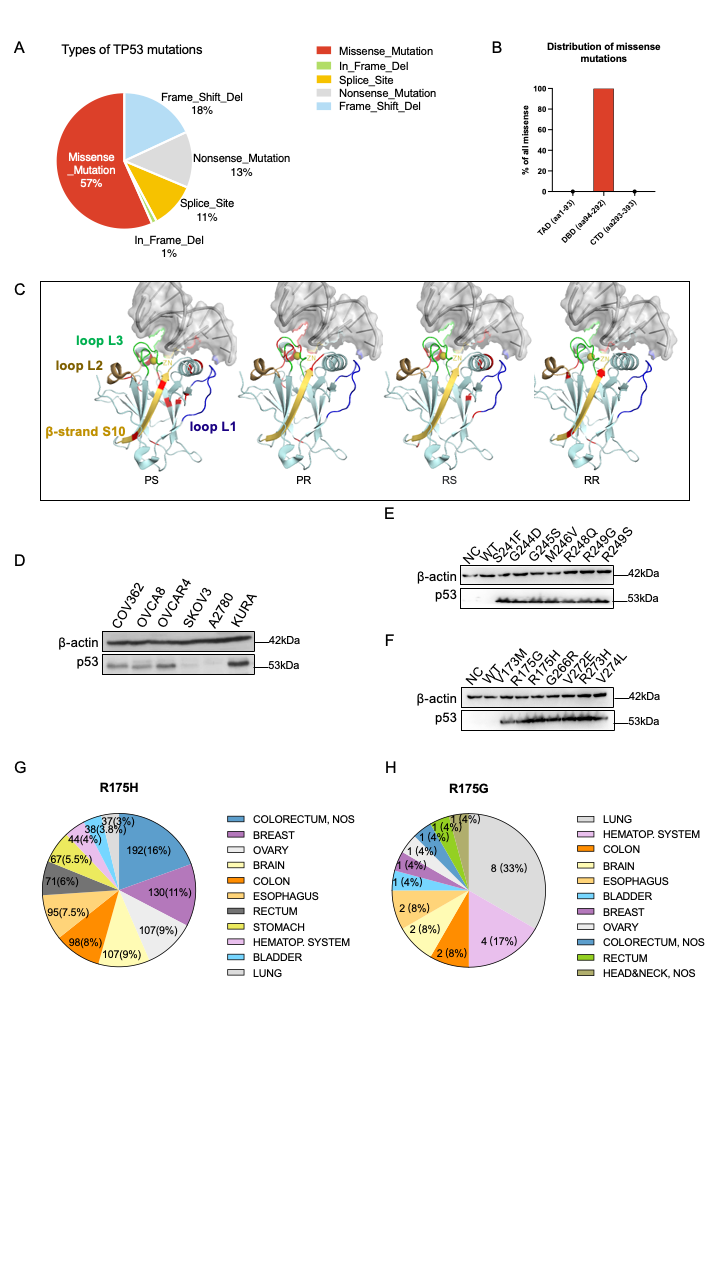
**

**Supplementary Figure 1**

**A** Proportion of TP53 mutation types identified in the study, with missense mutations comprising 57% of all detected mutations. **B** Distribution of TP53 missense mutations. **C** Structure of p53 bound to DNA (PDB entry 2AHI), with TP53 missense mutations highlighted in red. The L1, L2, L3, and S10 regions, critical for DNA binding, are marked in green, brown, blue, and yellow, respectively. **D** WB assay showing p53 expression levels in various ovarian cancer cell lines. **E****–F** TP53 missense mutations associated with regions of primary resistance or recurrent resistance were introduced into the p53-null SKOV3 cell line. **G–H** Mutation frequency of R175H and R175G across various cancers, as reported in the National Cancer Institute Database.

**
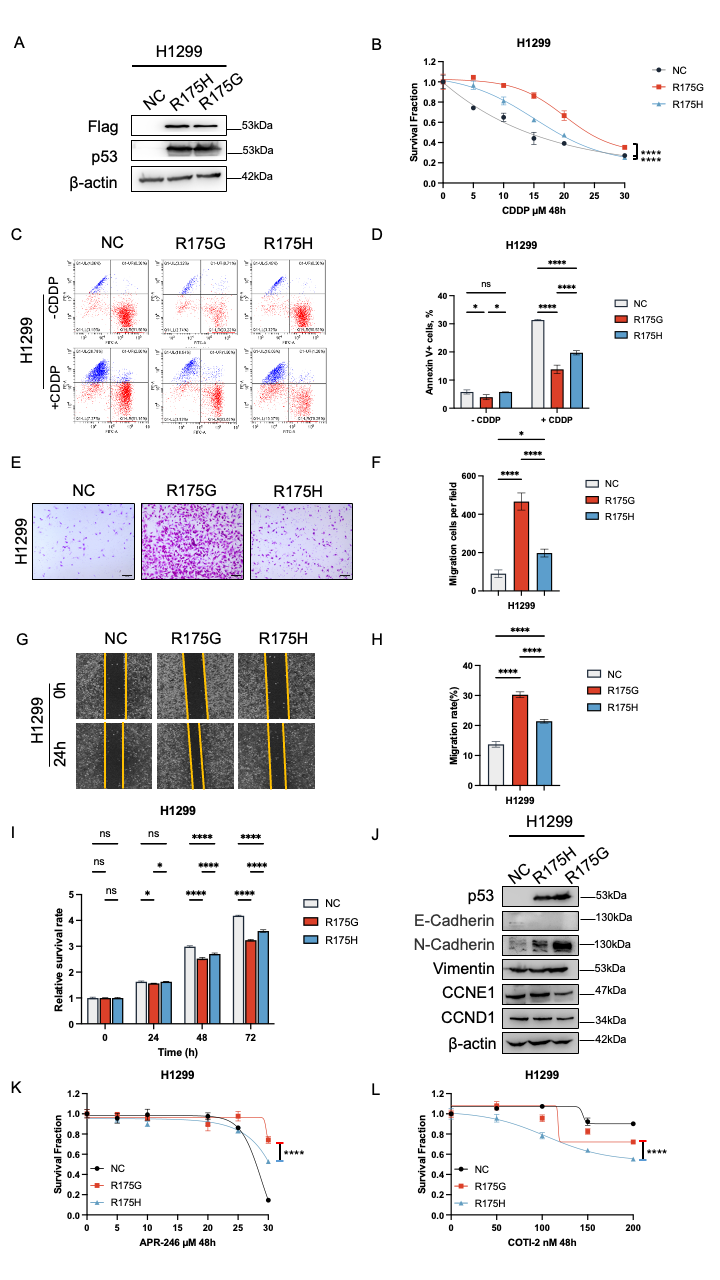
**

**Supplementary Figure 2**

**A** WB assay validating the successful establishment of p53-null (p53^NC^), p53^R175H^, and p53^R175G^ mutations in TP53-null H1299 cells. **B** CCK-8 assay validating the cisplatin resistance of p53^NC^, p53^R175G^ and p53^R175H^ in H1299 cells. The data are presented as a nonlinear fit. **C–D** Apoptosis assay validating the cisplatin-induced apoptosis of p53^NC^, p53^R175G^ and p53^R175H^ in H1299 cells. **E-F** Cell migration assay validating the cell migration capacity of p53^R175G^ and p53^R175H^ in H1299 cells. **I–J** Wound healing assay validating the wound healing rate of p53^NC^, p53^R175G^ and p53^R175H^ in H1299 cells. **I** CCK-8 assay validating the proliferation of p53^NC^, p53^R175G^ and p53^R175H^ in H1299 cells. **J** WB assay validating the expression levels of E-Cadherin, N-Cadherin, Vimentin, cyclin E1 (CCNE1) and cyclin D1 (CCND1) of p53^NC^, p53^R175G^ and p53^R175H^ in H1299 cells. **K–L** CCK-8 assay validating the sensitivity of p53^NC^, p53^R175G^ and p53^R175H^ to p53^R175H^-targeting drugs APR-246 and COTI-2. The data are presented as mean ± standard deviation: **P* < 0.05, ***P* < 0.01, ****P* < 0.001, *****P* < 0.0001, significant difference; ns, no significant difference.


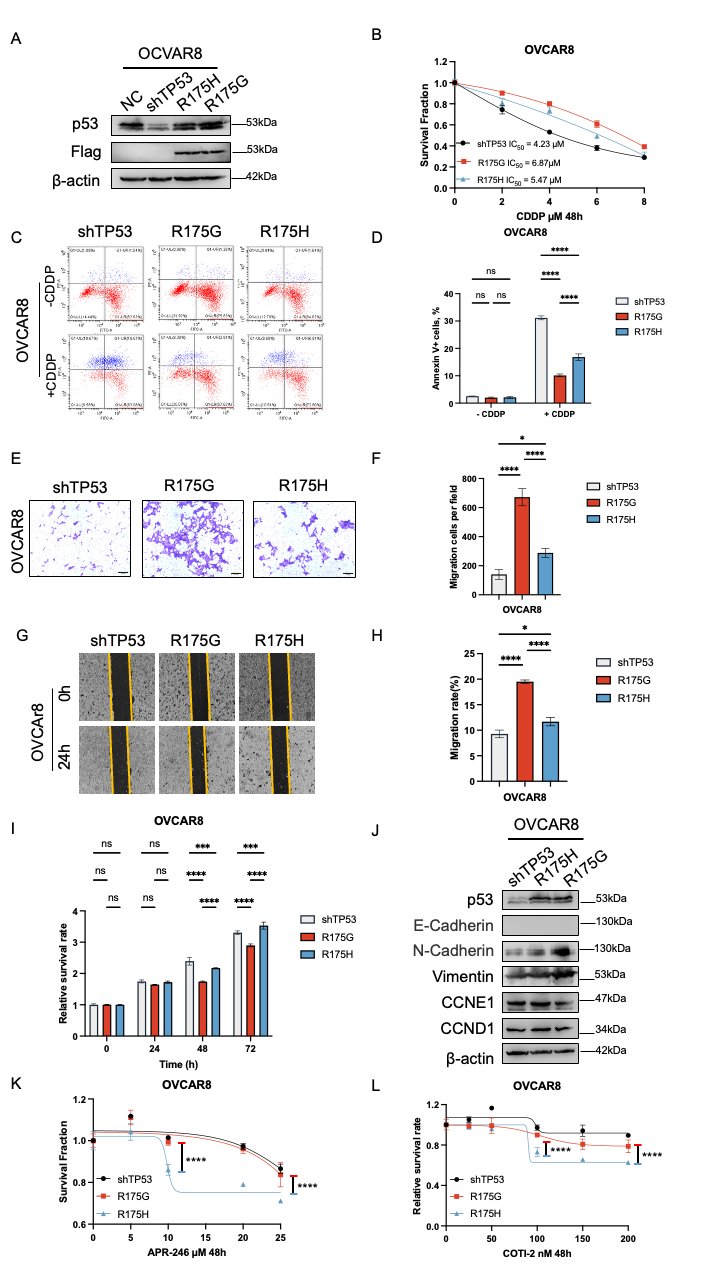


**Supplementary Figure 3**

**A** WB assay validating the successful establishment of p53^shTP53^, p53^R175H^, and p53^R175G^ mutations in OVCAR8 cells. **B** CCK-8 assay validating the cisplatin resistance of p53^shTP53^, p53^R175G^ and p53^R175H^ in OVCAR8 cells. The data are presented as a nonlinear fit. **C–D** Apoptosis assay validating the cisplatin-induced apoptosis of p53^shTP53^, p53^R175G^ and p53^R175H^ in OVCAR8 cells. **E-F** Cell migration assay validating the cell migration capacity of p53^R175G^ and p53^R175H^ in OVCAR8 cells. **I–J** Wound healing assay validating the wound healing rate of p53^shTP53^, p53^R175G^ and p53^R175H^ in OVCAR8 cells. **I** CCK-8 assay validating the proliferation of p53^shTP53^, p53^R175G^ and p53^R175H^ in OVCAR8 cells. **J** WB assay validating the expression levels of E-Cadherin, N-Cadherin, Vimentin, cyclin E1 (CCNE1) and cyclin D1 (CCND1) of p53^shTP53^, p53^R175G^ and p53^R175H^ in OVCAR8 cells. **K–L** CCK-8 assay validating the sensitivity of p53^shTP53^, p53^R175G^ and p53^R175H^ to p53^R175H^-targeting drugs APR-246 and COTI-2. The data are presented as mean ± standard deviation: **P* < 0.05, ***P* < 0.01, ****P* < 0.001, *****P* < 0.0001, significant difference; ns, no significant difference.


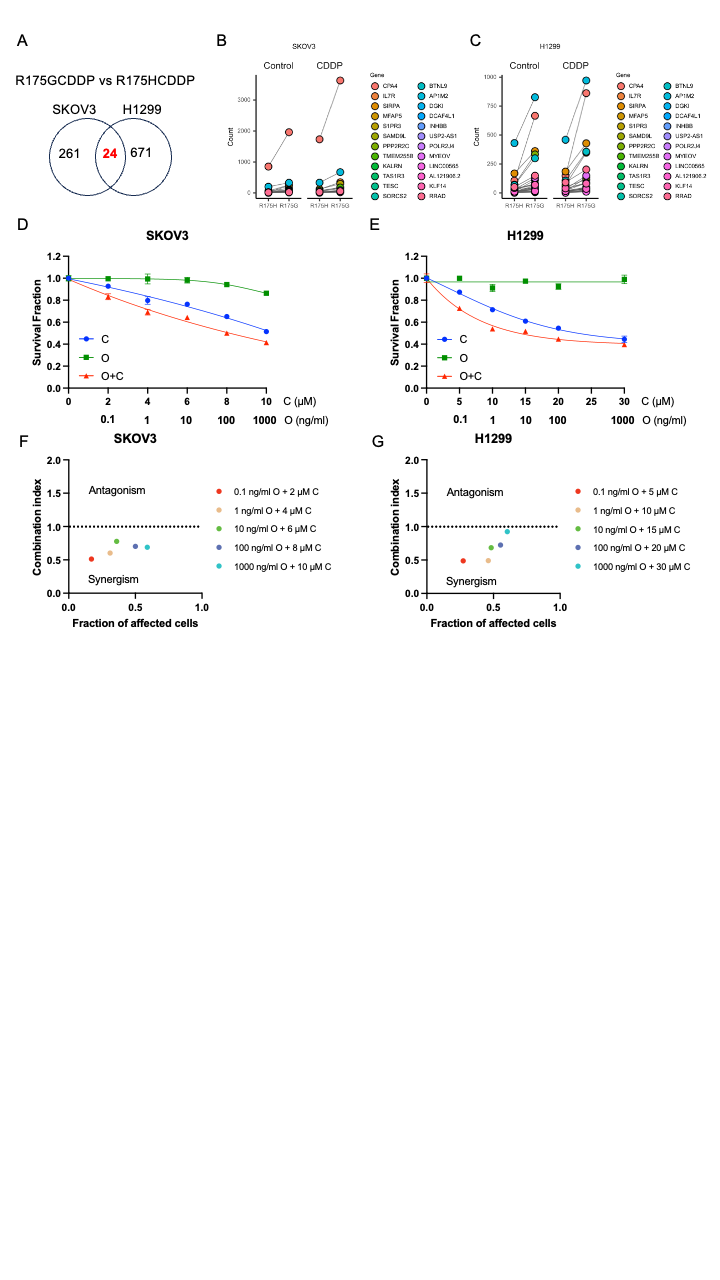


**Supplementary Figure 4**

**A** Significant DEGs of R175GCDDP compared to R175HCDDP in SKOV3 and H1299 cells (fold change ≥ 2, padj < 0.05). **B–C** Transcriptomic analysis revealed differential mRNA expression of DEGs in p53^R175H^ and p53^R175G^ in SKOV3 and H1299 cells following cisplatin treatment. DEGs were identified with padj < 0.05 and fold change ≥ 2. **D–E** SKOV3 and H1299 cells were treated with varying concentrations of OSE-127 (O) and cisplatin (C) for 48 hours, either alone or in combination. The survival fraction (SF) was assessed using the CCK-8 assay. Data are represented as mean ± SD (n = 3). **F–G** Combination index (CI) was determined using the Chou-Talalay method, where CI < 1 indicated synergy, CI = 1 indicated additivity, CI > 1 indicated antagonism.


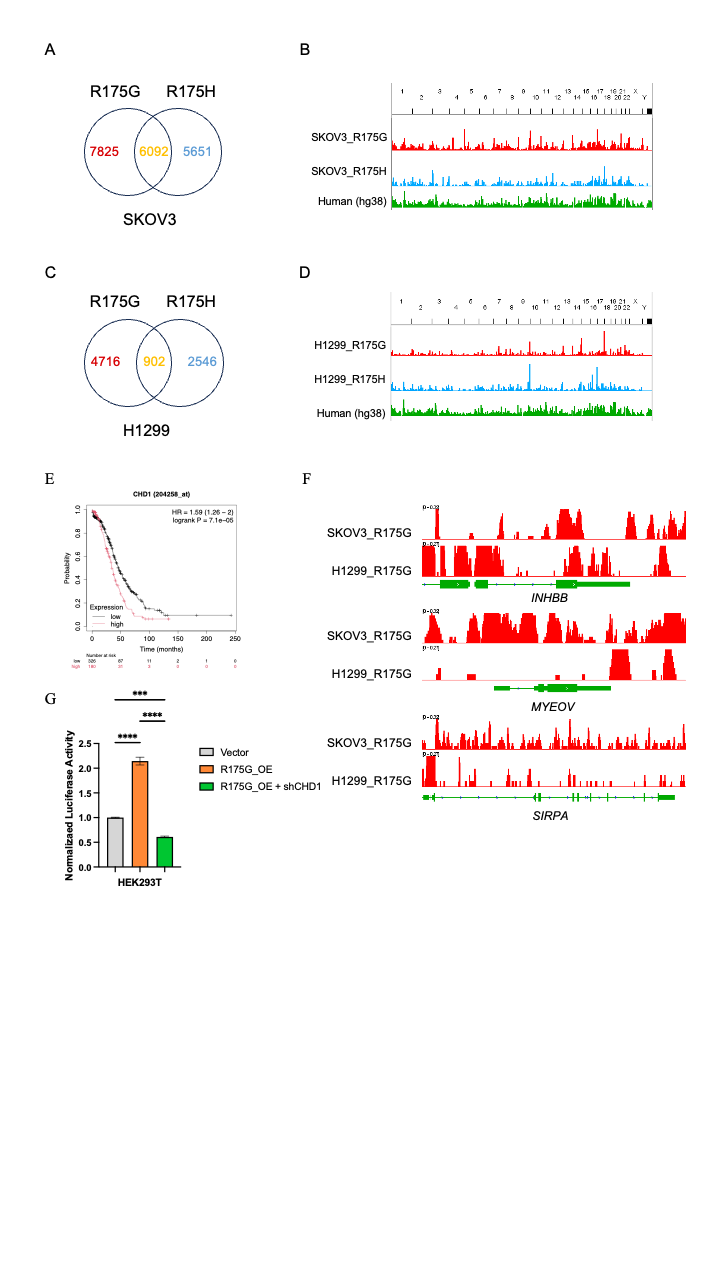


**Supplementary Figure 5**

**A** Wayne diagram showing genes bound by p53^R175G^ and p53^R175H^ in SKOV3 cells. **B** Distribution of reads across the genome in SKOV3 cells. **C** Wayne diagram showing genes bound by p53^R175G^ and p53^R175H^ in H1299 cells. **D** Distribution of reads across the genome in H1299 cells. **E** Kaplan-Meier survival analysis comparing CHD1 expression (high vs. low) in ovarian cancer patients carrying TP53 mutations, showing overall survival outcomes. Data were generated using Kaplan-Meier Plotter (n = 326 [low] and n = 180 [high]; false discovery rate [FDR] = 1%). **F** ChIP-seq signals for p53^R175G^ target genes, including *INHBB*, *MYEOV*, and *SIRPA* in SKOV3 and H1299 cells. **G** HEK293T cells overexpressing Vector, p53^R175G^, or p53^R175G^ co-transfected with CHD1 siRNA for 48 h and then subjected to a dual luciferase reporter assay. All the experiments were performed in triplicate and data are presented as the mean ± SD using an ordinary one-way ANOVA test; ****P* < 0.001, *****P* < 0.0001.
