# Supplementary material for: Distinct functional heterogeneity of TP53 R175 mutations in platinum-resistant ovarian cancer: unveiling molecular mechanisms and therapeutic targets
Source: Cell Death Dis. 2025 Nov 17;16(1):837. doi: 10.1038/s41419-025-08172-0 (PMC12623820; doi:10.1038/s41419-025-08172-0)
Supplement: Supplementary file 2 — Supplementary Tables [file 41419_2025_8172_MOESM2_ESM.docx]

**Supplementary Tables**

**Table 1****.** Fisher exact test (L3, aa240-250)

| Category | OthMut | Mut | Total | Within-group Percentage |  |
| --- | --- | --- | --- | --- | --- |
| PS  PR  p.result | 14  8 | 0  6 | 14  14 | OthMut: 100.0%; Mut: 0.0%  WT: 57.1%; Mut: 42.9%  p = 0.016 |  |

**Table 2.** Fisher exact test (L2, S10)

| Category | OthMut | Mut | Total | Within-group Percentage |  |
| --- | --- | --- | --- | --- | --- |
| RS  RR  p.result | 10  3 | 1  5 | 11  8 | OthMut: 90.91%; Mut: 9.09%  OthMut: 37.5%; Mut: 62.5%  p = 0.041 |  |

**Table 3.** Cisplatin resistance-associated mutant domains

| Domains | Amino acid |  |
| --- | --- | --- |
| L3 (aa240-250)  L2/S10 | S241F, G244D, G245S, M246V, R248Q, R249G, and R249S  V173M, R175G, R175H, G266R, V272E, R273H, and V274L |  |

**Table 4.** TP53 mutations in ovarian cancer cell lines

| **Cell Line** | **Disease Subtype** | **Variant Classification** | **Variant Type** | **Protein Change** |
| --- | --- | --- | --- | --- |
| COV362  Kuramochi  OVCAR4  OVCAR8  SKOV3 | Adenocarcinoma, high grade serous  Adenocarcinoma, high grade serous  Adenocarcinoma, high grade serous  Adenocarcinoma, high grade serous  Cystadenocarcinoma, endometrioid | Missense_Mutation  Missense_Mutation  Missense_Mutation  Splice_Site  Frame_Shift_Del | SNP  SNP  SNP  SNP  DEL | p.Y220C  p. D281Y  p. L130V  NA  p. P89fs |
| A2780 | Cystadenocarcinoma, endometrioid | WT | NA | NA |

**Table 5.** One-way ANOVA was employed to analyze the differences in CDDP IC_50_ between the mutation groups (L3, aa240-250) and the NC group

| Test details | Mean 1 | Mean 2 | Mean Diff. | 95.00% CI of diff. | Adjusted P Value | Summary |
| --- | --- | --- | --- | --- | --- | --- |
| NC vs. WT | 1.546 | 2.62 | -1.074 | -1.390 to -0.7579 | <0.0001 | **** |
| NC vs. S241F | 1.546 | 5.039 | -3.493 | -3.810 to -3.177 | <0.0001 | **** |
| NC vs. G244D | 1.546 | 2.815 | -1.27 | -1.586 to -0.9535 | <0.0001 | **** |
| NC vs. G245S | 1.546 | 4.482 | -2.937 | -3.253 to -2.621 | <0.0001 | **** |
| NC vs. M246V | 1.546 | 2.589 | -1.043 | -1.360 to -0.7272 | <0.0001 | **** |
| NC vs. R248Q | 1.546 | 5.337 | -3.791 | -4.107 to -3.475 | <0.0001 | **** |
| NC vs. R249G | 1.546 | 4.057 | -2.511 | -2.827 to -2.195 | <0.0001 | **** |
| NC vs. R249S | 1.546 | 4.523 | -2.977 | -3.294 to -2.661 | <0.0001 | **** |

**Table 6.** One-way ANOVA was employed to analyze the differences in CDDP IC_50_ between the mutation groups (L3, aa240-250) and the WT group

| Test details | Mean 1 | Mean 2 | Mean Diff. | 95.00% CI of diff. | Adjusted P Value | Summary | |
| --- | --- | --- | --- | --- | --- | --- | --- |
| WT vs. NC | 2.62 | 1.546 | 1.074 | 0.7579 to 1.390 | <0.0001 | | **** |
| WT vs. S241F | 2.62 | 5.039 | -2.419 | -2.735 to -2.103 | <0.0001 | | **** |
| WT vs. G244D | 2.62 | 2.815 | -0.1956 | -0.5117 to 0.1206 | 0.3671 | | ns |
| WT vs. G245S | 2.62 | 4.482 | -1.863 | -2.179 to -1.546 | <0.0001 | | **** |
| WT vs. M246V | 2.62 | 2.589 | 0.03072 | -0.2855 to 0.3469 | >0.9999 | | ns |
| WT vs. R248Q | 2.62 | 5.337 | -2.717 | -3.033 to -2.401 | <0.0001 | | **** |
| WT vs. R249G | 2.62 | 4.057 | -1.437 | -1.753 to -1.121 | <0.0001 | | **** |
| WT vs. R249S | 2.62 | 4.523 | -1.903 | -2.220 to -1.587 | <0.0001 | | **** |

**Table 7.** One-way ANOVA was employed to analyze the differences in CDDP IC_50_ between mutations groups (L2, S10) and NC group.

| Test details | Mean 1 | Mean 2 | Mean Diff. | 95.00% CI of diff. | Adjusted P Value | Summary |
| --- | --- | --- | --- | --- | --- | --- |
| NC vs. WT | 1.546 | 2.62 | -1.074 | -1.694 to -0.4539 | 0.0005 | *** |
| NC vs. V173M | 1.546 | 4.17 | -2.625 | -3.245 to -2.004 | <0.0001 | **** |
| NC vs. R175G | 1.546 | 11.64 | -10.1 | -10.72 to -9.476 | <0.0001 | **** |
| NC vs. R175H | 1.546 | 5.881 | -4.335 | -4.955 to -3.715 | <0.0001 | **** |
| NC vs. G266R | 1.546 | 4.823 | -3.277 | -3.898 to -2.657 | <0.0001 | **** |
| NC vs. V272E | 1.546 | 3.678 | -2.133 | -2.753 to -1.513 | <0.0001 | **** |
| NC vs. R273H | 1.546 | 4.509 | -2.964 | -3.584 to -2.343 | <0.0001 | **** |
| NC vs. V274L | 1.546 | 3.132 | -1.586 | -2.207 to -0.9662 | <0.0001 | **** |

**Table 8.** One-way ANOVA was employed to analyze the differences in CDDP IC_50_ between mutations groups (L2, S10) and WT group.

| Test details | Mean 1 | Mean 2 | Mean Diff. | 95.00% CI of diff. | Adjusted P Value | Summary |
| --- | --- | --- | --- | --- | --- | --- |
| WT vs. NC | 2.62 | 1.546 | 1.074 | 0.4539 to 1.694 | 0.0005 | *** |
| WT vs. V173M | 2.62 | 4.17 | -1.551 | -2.171 to -0.9303 | <0.0001 | **** |
| WT vs. R175G | 2.62 | 11.64 | -9.022 | -9.642 to -8.402 | <0.0001 | **** |
| WT vs. R175H | 2.62 | 5.881 | -3.261 | -3.881 to -2.641 | <0.0001 | **** |
| WT vs. G266R | 2.62 | 4.823 | -2.203 | -2.824 to -1.583 | <0.0001 | **** |
| WT vs. V272E | 2.62 | 3.678 | -1.059 | -1.679 to -0.4385 | 0.0006 | *** |
| WT vs. R273H | 2.62 | 4.509 | -1.889 | -2.510 to -1.269 | <0.0001 | **** |
| WT vs. V274L | 2.62 | 3.132 | -0.5123 | -1.132 to 0.1079 | 0.1333 | ns |

**Table 9.** TP53 mutations primers

| S241F | 5'-ATGTGTAACAGTTTCTGCATGGGCGG-3' | 5'-CCGCCCATGCAGAAACTGTTACACAT-3' |
| --- | --- | --- |
| G244D | 5'-TTCCTGCATGGACGGCATGAAC-3' | 5'-GTTCATGCCGTCCATGCAGGAA-3' |
| G245S | 5'-TTCCTGCATGGGCAGCATGAACCGGAGG-3' | 5'-CCTCCGGTTCATGCTGCCCATGCAGGAA-3' |
| M246V | 5'-CATGGGCGGCGTGAACCGGAGG-3' | 5'-CCTCCGGTTCACGCCGCCCATG-3' |
| R248Q | 5'-CGGCATGAACCAGAGGCCCATCCT-3' | 5'-AGGATGGGCCTCTGGTTCATGCCG-3' |
| R249G | 5'-GCATGAACCGGGGGCCCATCCTC-3' | 5'-GAGGATGGGCCCCCGGTTCATGC-3' |
| R249S | 5'-CGGCATGAACCGGAGCCCCATCCTCA-3' | 5'-TGAGGATGGGGCTCCGGTTCATGCCG-3' |
| V173M | 5'-ATGACGGAGGTTATGAGGCGCTG-3' | 5'-CAGCGCCTCATAACCTCCGTCAT-3' |
| R175G | 5'-GGTGGGGGCAGCCCCTCACAACTTC-3' | 5'-GAGGTTGTGAGGGGCTGCCCACC-3' |
| R175H | 5'-ATGGTGGGGGCAGTGCCCTCACAACCTC-3' | 5'-GAGGTTGTGAGGCACTGCCCACCAT-3' |
| G266R | 5'-CTCAAAGCTGTTCCGTCTCAGTAGATTACCACTGG-3' | 5'-CCAGTGGTAATCTACTGAGACGGAACAGCTTTGAG-3' |
| V272E | 5'-AACAGCTTTGAGGAGCGTGTTTGTGC-3' | 5'-GCACAAACACGCTCCTCAAAGCTGTT-3' |
| R273H | 5'-CTTTGAGGTGCATGTTTGTGCCTGT-3' | 5'-ACAGGCACAAACATGCACCTCAAAG-3' |
| V274L | 5'-TTTGAGGTGCGTCTTTGTGCCTGTCCT-3' | 5'-AGGACAGGCACAAAGACGCACCTCAAA-3' |

**Table 10.** siRNA primers

| shCHD1 | 5'-CCGGGCGGTTTATCAAGAGCTATAACTCGAGTTATAGCTCTTGATAAACCGCTTTTTG-3' | 5'-AATTCAAAAAGCGGTTTATCAAGAGCTATAACTCGAGTTATAGCTCTTGATAAACCGC-3' |
| --- | --- | --- |

**Table 10.** ChIP-qPCR primers

| IL7R-1 | 5'-TTCTAATGACTAACTCAAAGTC-3' | 5'-GACTTTGAGTTAGTCATTAGAA-3' |
| --- | --- | --- |
| IL7R-2 | 5'-TGTTCTTTACCTTTTGTAGA-3' | 5'-TCTACAAAAGGTAAAGAACA-3' |
| IL7R-3 | 5'-TTTGGTCCAACAAACATGAC-3' | 5'-GTCATGTTTGTTGGACCAAA-3' |
| GAPDH | 5'-GCACGTAGCTCAGGCCTCAAGAC-3' | 5'-GTCTTGAGGCCTGAGCTACGTGC-3' |

**Table 11.** qRT-PCR primers

| GAPDH | 5'-CTGGGCTACACTGAGCACC-3' | 5'-AAGTGGTCGTTGAGGGCAATG-3' |
| --- | --- | --- |
| IL7R | 5'-CAGCAATGTATGAGATTA-3' | 5'-ATGGTTAGTAAGATAGGAT-3' |
| DGKI | 5'-ATGACATCCATCAGGTGCAA-3' | 5'-CCGGAGTTTGTCCTTGTCAT-3' |
| SIRPA | 5'-TTCCAGTGCCTTCCAGCCCT-3' | 5'-GGTGATGTTACCGATGCGGATG-3' |
| MFAP5 | 5'-ATGTCGCTCTTGGGACC-3' | 5'-TCACAGACCATTGGGTCTC-3' |
| SORCS2 | 5'-CAAGGAAGAGGAGCCTCTCGAGTGATA-3' | 5'-GTGGTTCTGTGCCCTCCGTGGGTGAAA-3' |
| AP1M2 | 5'-TGTCTTCATTCTGGACGTTAAGG-3' | 5'-AGTGCTCAATCTTGCTCATGG-3' |
| INHBB | 5'-ATCAGCTTCGCCGAGACA-3' | 5'-GGTTGCCTTCGTTGGAGAT-3' |
| MYEOV | 5'-CCTAAATCCAGCCACGTCAT-3' | 5'-GACACACCACGGAGACAATG-3' |
