# Supplementary material for: Distinct functional heterogeneity of TP53 R175 mutations in platinum-resistant ovarian cancer: unveiling molecular mechanisms and therapeutic targets
Source: Cell Death Dis. 2025 Nov 17;16(1):837. doi: 10.1038/s41419-025-08172-0 (PMC12623820; doi:10.1038/s41419-025-08172-0)

Fig.2A

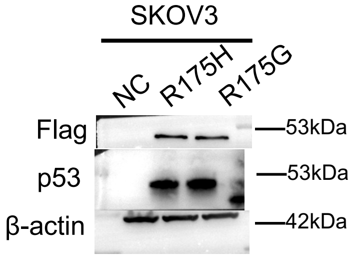

Fig.2J

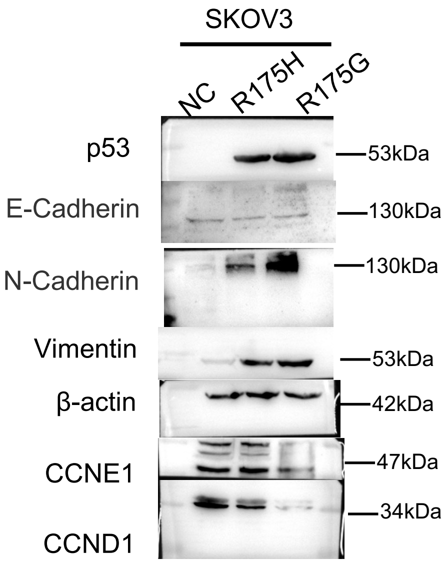

Fig.5 A

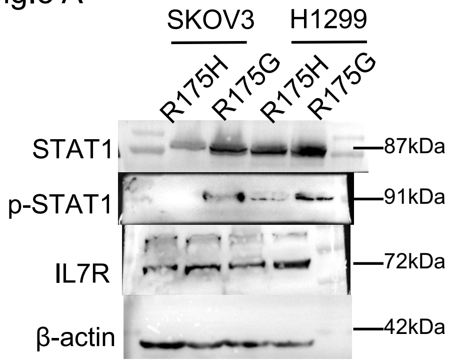

Fig.5 J

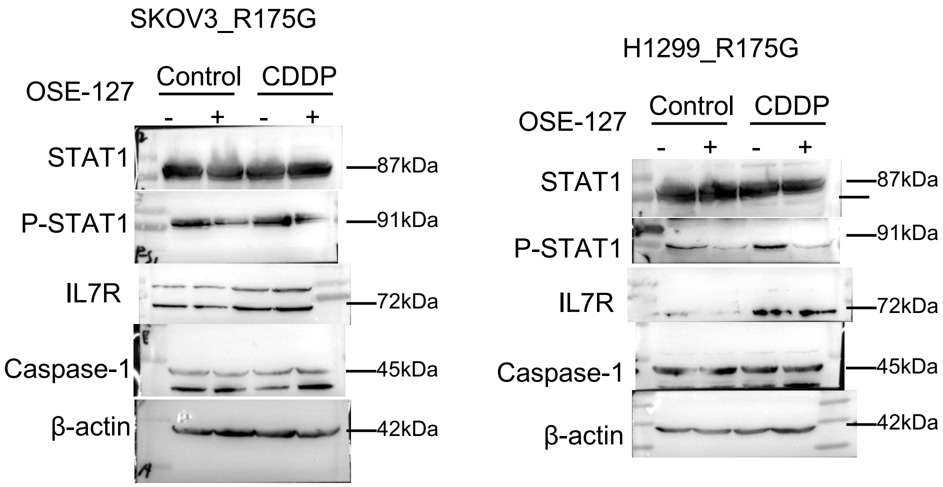

Fig.7 F

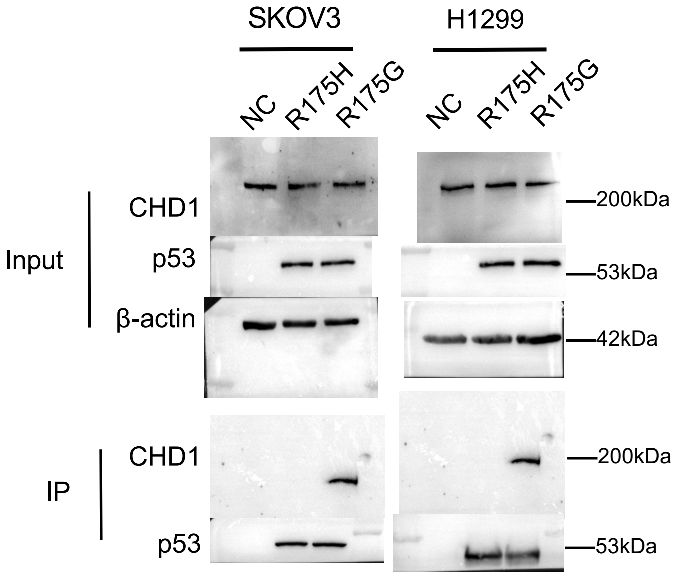

Fig.7 G

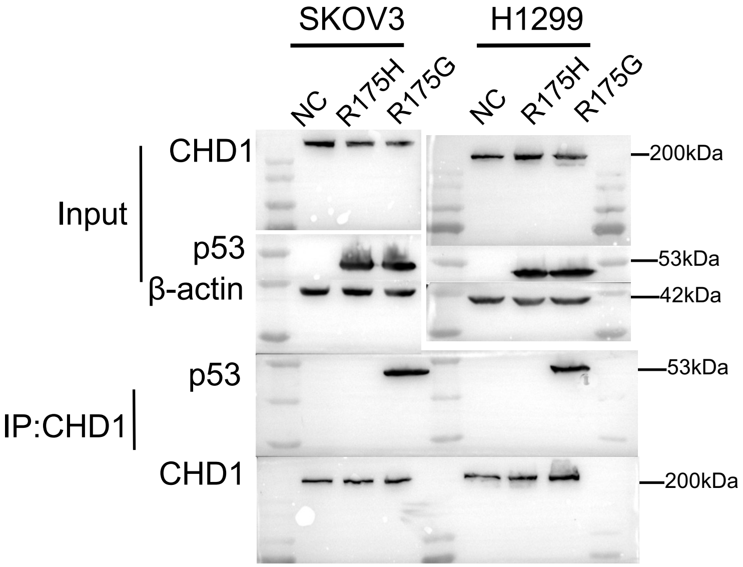

Fig.7 H

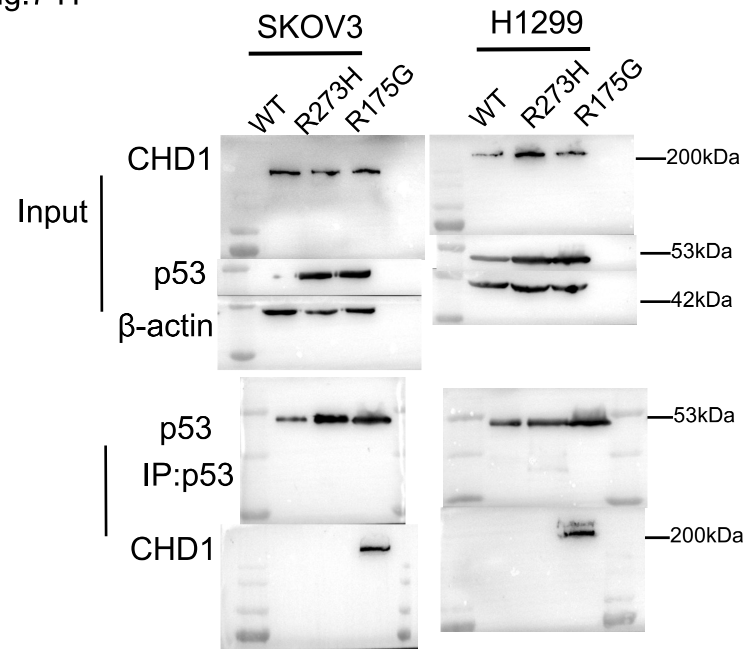

Fig.8 A

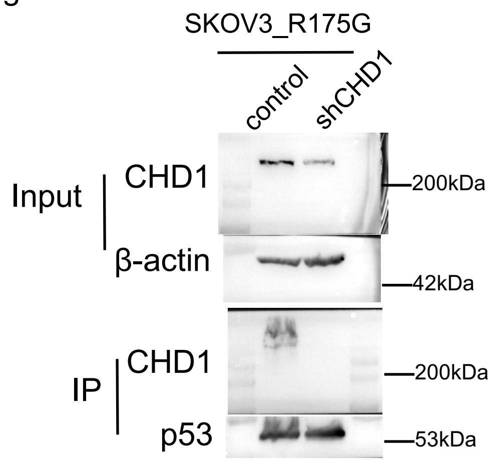

Fig.8 B

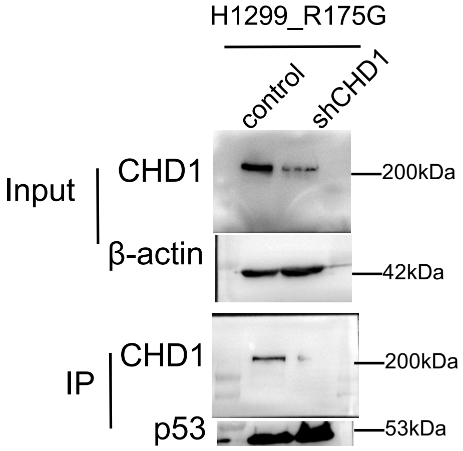

Fig.8 E

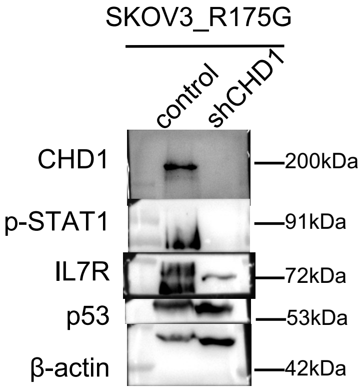

Fig.8 F

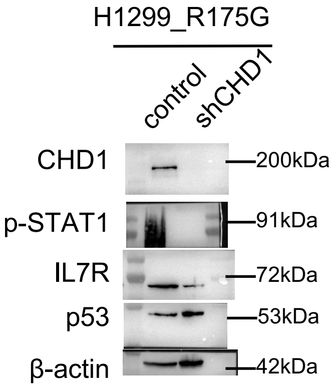

Supplementary. Fig.1D

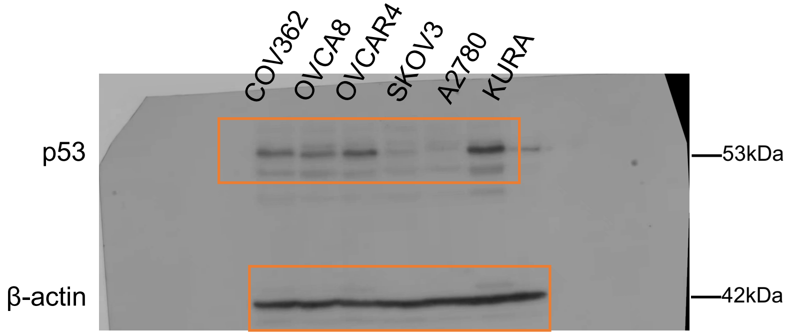

Supplementary. Fig.1E

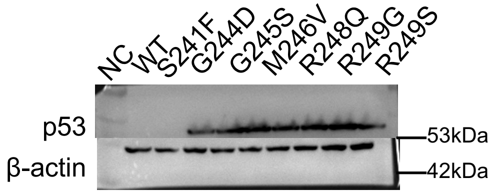

Supplementary. Fig.1F

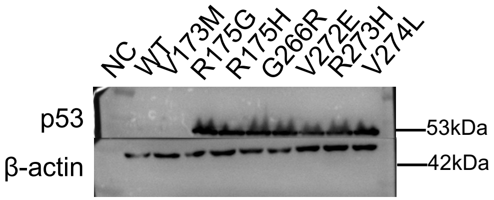

Supplementary. Fig.2A

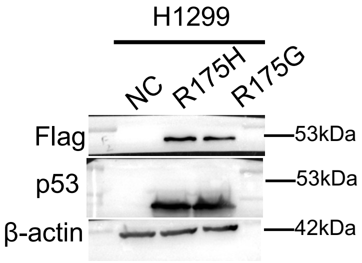

Supplementary. Fig.2J

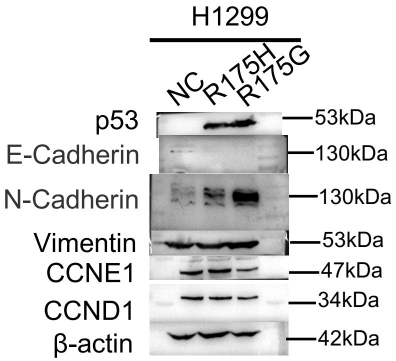

Supplementary. Fig.3A

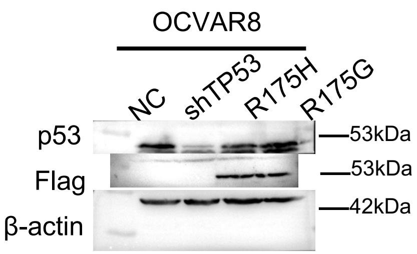

Supplementary. Fig.3J

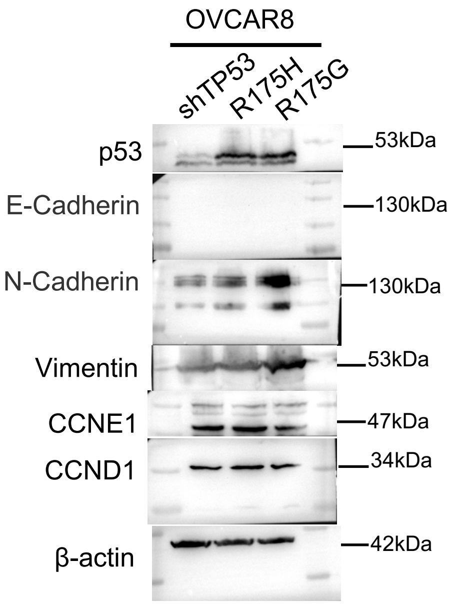

Supplement: Supplementary file 3 — original western blot data [file 41419_2025_8172_MOESM3_ESM.pdf]
